# Seasonal habitat-use patterns of large mammals in a human-dominated landscape

**DOI:** 10.1093/jmammal/gyad107

**Published:** 2023-11-24

**Authors:** Dilsad Dagtekin, Alper Ertürk, Stefan Sommer, Arpat Ozgul, Anil Soyumert

**Affiliations:** Department of Evolutionary Biology and Environmental Studies, University of Zurich, Winterthurerstrasse 190, CH-8057 Zurich, Switzerland; Hunting and Wildlife Program, Araç Rafet Vergili Vocational School of Higher Education, Kastamonu University, TR-37800, Arac, Kastamonu, Turkey; Department of Evolutionary Biology and Environmental Studies, University of Zurich, Winterthurerstrasse 190, CH-8057 Zurich, Switzerland; Department of Evolutionary Biology and Environmental Studies, University of Zurich, Winterthurerstrasse 190, CH-8057 Zurich, Switzerland; Hunting and Wildlife Program, Araç Rafet Vergili Vocational School of Higher Education, Kastamonu University, TR-37800, Arac, Kastamonu, Turkey

**Keywords:** Bayesian, camera trap, hierarchical models, imperfect detection, large mammals, occupancy modeling, seasonality

## Abstract

Large mammals in temperate climates typically display seasonal patterns of habitat use. However, these patterns are often overlooked because large mammals are usually surveyed at annual intervals. In addition, most studies focus on a single species and ignore other species with which the focal species could interact. Knowing seasonal patterns of habitat use in multiple species and understanding factors that cause these patterns can provide further detail on population dynamics and guide effective conservation planning. Here, using dynamic occupancy modeling, we analyze 11 years of camera-trap data collected in northwestern Anatolia, Turkey, to investigate seasonal habitat use of 8 large-mammal species: Brown Bear (*Ursus arctos*), Eurasian Lynx (*Lynx lynx*), Gray Wolf (*Canis lupus*), Red Fox (*Vulpes vulpes*), Wild Boar (*Sus scrofa*), Roe Deer (*Capreolus capreolus*), European Hare (*Lepus europaeus*), and Red Deer (*Cervus elaphus*). For each species, we study the strength of seasonality in habitat use and its dependence on human population density and elevation, which have been shown to affect distributions of species in the region. Although all species exhibited seasonality in habitat use, the strength of this seasonality varied among species; it was strongest in Wild Boar, Roe Deer, and Brown Bear. Moreover, except for Brown Bear, all species tended to avoid sites close to humans. The species responded differently to changing elevation; increasing elevation had both positive and negative effects on species-specific colonization and desertion probabilities, and these effects were likely related to either feeding habits or tendency to avoid humans. These results indicate that seasonality should be taken into consideration in population studies. However, because species differ, seasonality patterns should be identified separately for each species of interest, as differences in these patterns can explain the underlying dynamics of habitat-use patterns more accurately.

## Teaser

How does environmental seasonality affect habitat use of 8 large-mammal species in northwestern Anatolia, Turkey? We show that the strength of seasonality in habitat use varies among the species. These variations have direct implications for conservation.

Many wildlife populations inhabit environments in which natural resources, human activities, and climate characteristics including rainfall and temperature vary seasonally. Such environmental seasonality causes seasonal patterns in the habitat use and population dynamics of species (Taylor et al. 2013). Although environmental seasonality has long been recognized as a major factor in life-history adaptations, wildlife data are often collected at annual intervals or at specific times of the year (e.g. when the study species is breeding or the study site is easily accessible). Such studies can miss important within-year changes in environmental factors and, consequently, population responses to these changes (White and Hastings 2020). Additionally, global climate change and other anthropogenic environmental changes often affect some seasons more than others (Taylor et al. 2013; Smeraldo et al. 2018; Zurell et al. 2018). In these cases, if environmental and population data are analyzed at annual steps, changes in seasonal responses can remain undetected (Varpe 2017).

Studying wildlife populations year-round provides insight into how survival, recruitment, and growth rates (e.g. Woodroffe et al. 2017; Paniw et al. 2019) and habitat-use patterns (e.g. Lafferty 2001; Nielsen et al. 2010; Fourcade et al. 2018) vary seasonally. Seasonal changes in habitat-use patterns are common, because important resources including food and shelter often vary seasonally, affecting the distribution of many species (Stein et al. 2014). Understanding how and why species distributions change with seasons is important, because such understanding can inform future population studies and conservation actions by revealing mechanisms of population dynamics (Chuine 2010; Forrest and Miller-Rushing 2010). Environmental seasonality can constrain energy acquisition and, as a result, lower survival and reproduction by making some habitats less suitable at certain times of the year (Varpe 2017). Species cope with these constraints by adopting seasonal strategies, often through mechanisms of habitat selection. For example, they might breed only in a specific habitat at a certain time of the year (e.g. Fourcade et al. 2018), migrate seasonally (e.g. Coppes et al. 2017), or—to avoid harsh conditions without migrating—go into torpor or hibernate (e.g. Paniw et al. 2020).

Here, we study seasonal habitat-use patterns of 8 medium- to large-sized mammal species (for convenience, hereafter referred to as “large mammals”) in a human-dominated region in northwestern Anatolia, Turkey. Northwestern Anatolia is rich in native large-mammal species and therefore considered a globally important region for wildlife (Morrison et al. 2007). The region is highly seasonal with marked differences in temperature and precipitation between summer and winter, with most precipitation falling during early summer (climate-data.org 2021). As a result, many human activities (e.g. hunting, forestry, and agriculture) are also seasonal. Large mammals in the region have been studied with respect to daily activity patterns and distribution ranges (Soyumert 2010). However, whether and how seasonality affects habitat use and how species differ in their seasonal habitat use are still unknown. We aim to fill these knowledge gaps by using camera-trap data to identify common features in seasonal habitat-use patterns and differences in the strength of seasonality in these patterns.

## Materials and methods

### Study species and area.

**Fig. 1.**
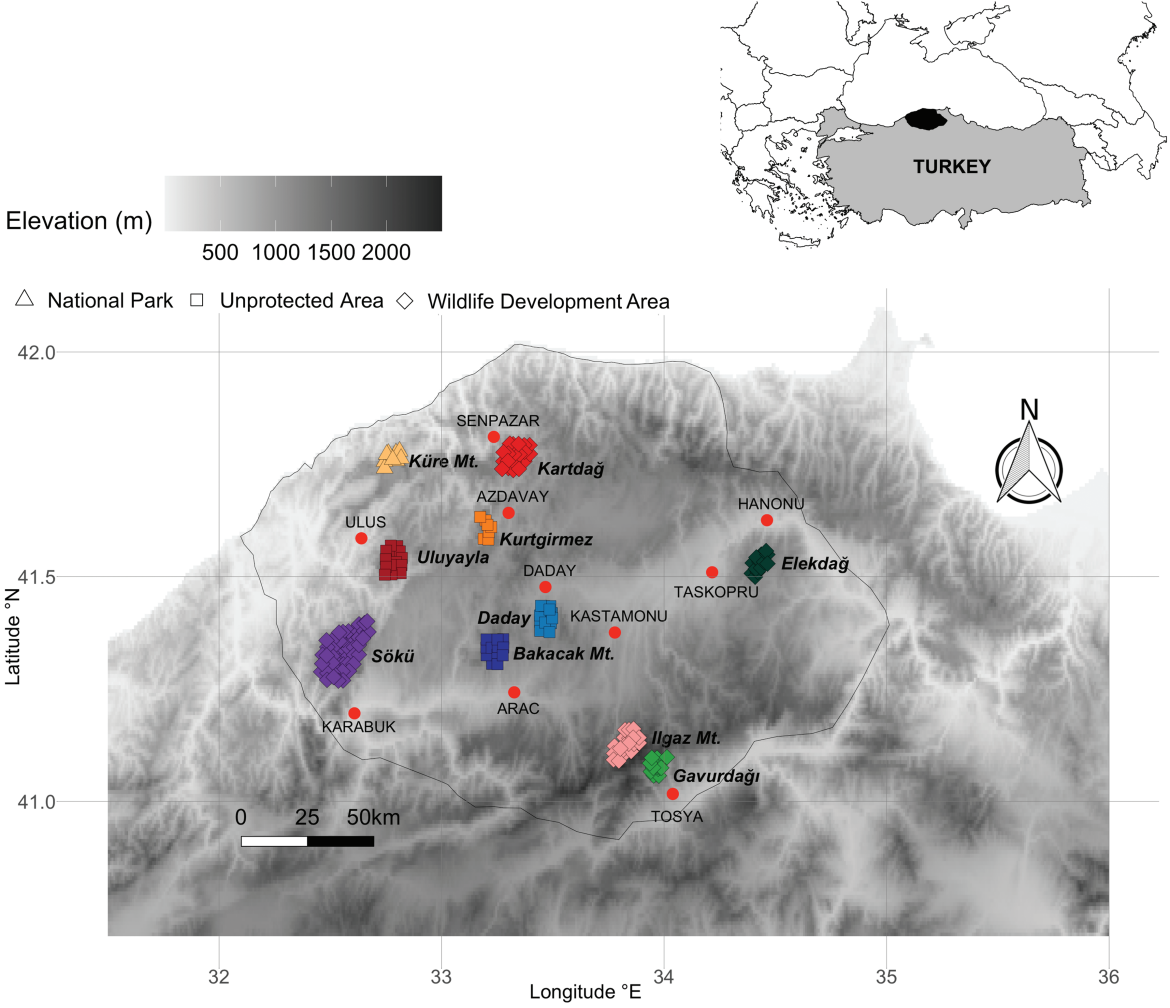
Locations of the study area (top-right, black area) and camera traps (bottom) in a national park (colored triangles), 4 unprotected areas (colored squares), and 5 wildlife developmental areas (colored diamonds). The clusters of camera traps represent the 10 study sites; red dots indicate major settlements closest to these sites.

We studied habitat-use patterns of 4 predator species; Brown Bear (*Ursus arctos*), Eurasian Lynx (*Lynx lynx*), Gray Wolf (*Canis lupus*), Red Fox (*Vulpes vulpes*)—and 4 prey species; Wild Boar (*Sus scrofa*), Roe Deer (*Capreolus capreolus*), European Hare (*Lepus europaeus*), and Red Deer (*Cervus elaphus*) at 10 sites in northwestern Anatolia, Turkey (42°01ʹ09″ to 40°54ʹ28″N; 32°05ʹ36″ to 34°40ʹ44″E; 20,738 km^2^; Fig. 1). Five of the sites are inside the wildlife development areas Sökü, Ilgaz Mountain, Kartdağ, and Gavurdağ, and 1 site is inside the Küre Mountains National Park. In these areas, wildlife populations are protected but controlled through hunting (e.g. Wild Boar, Roe Deer, and Red Deer). The remaining 4 sites are in unprotected areas. Elevation ranges over which mammal species are mostly found covers a gradient from sea level to 2,587 m a.s.l. at the peak of Mount Ilgaz. The region is dominated by forests composed of Oriental Beech (*Fagus orientalis*), Caucasian Fir (*Abies nordmanniana*), Black Pine (*Pinus nigra*), and Scots Pine (*Pinus sylvestris*).

According to the Köppen–Geiger climate classification, northwestern Anatolia has 2 types of climate—the temperate oceanic climate and the warm-summer, humid continental climate (Peel et al. 2007). This climate diversity causes seasonally varying temperature and precipitation regimes. To study the effects of this variation on habitat-use patterns, we divided the year into the seasons summer (May–October) and winter (December–March). To allow for seasonal transitions in the analysis, we removed the transition months April and November. This definition of seasons, 4-month winters and 6-month summers, is discrete and therefore may diffuse some seasonality effects in the analysis. While species-specific definitions of seasons might better reveal seasonal habitat-use patterns for each species, we prioritize a multispecies perspective and a replicable framework for further studies and possible conservation practices. Based on our definitions, average summer temperature and precipitation are 16.4 °C (10.9 to 20.2 °C) and 46.3 mm (30.4 to 75.2 mm); and average winter temperature and precipitation are 1.2 °C (−1.0 to 4.4 °C) and 31.4 mm (27.3 to 35.1 mm; Turkish State Meteorological Service 2021). In winter, the region is mostly covered in snow.

The study area is also affected by human activities that vary seasonally. Usually, local inhabitants leave the area before winter and return after snow melt to resume farming. Moreover, because forests are easier to access after snow melt, forestry activities peak in summer. The hunting season starts with the first snow at the beginning of winter, whereas poaching occurs more in summer. These different activities create a mosaic of open forests, shrublands, and agricultural fields with seasonally changing land-use dynamics.

### Camera-trap sampling.

We conducted systematic camera-trap surveys during 34 months from December 2007 to September 2010 and during 55 months from May 2014 to November 2018 at 10 sites equipped with 171 camera-trap stations (Fig. 1). We divided the study area into 2 × 2 km^2^ grid cells (adjusted to the smallest home-range size among the study species, 3 km^2^ for the European Hare; Soyumert 2010). Each grid cell contained a single, motion-triggered passive camera (Camtrakker, CamTrak South, Watkinsville, Georgia; Bushnell Trophy Cam HD, Bushnell Outdoor Products, Overland Park, Kansas). The cameras were placed along the most frequently used animal trails at the point closest to the center of a grid cell. We identified these trails in a preliminary study (Soyumert 2010) before starting the systematic survey. We placed the cameras at 50 to 60 cm above ground and recorded the coordinates and elevation of the stations. Because all large-mammal species were targeted, we did not use any bait or attractants. Motion triggers were active continuously. When activated, images were taken in triplets, with a 5-s delay between triggers. We checked the stations approximately every 1.5 months to download data and change batteries. We analyzed all images for species identity, recording date, and time.

### Environmental and anthropogenic covariates.

We used 3 environmental covariates and 1 anthropogenic covariate to study external effects on the occupancy and detection probabilities of each species. Environmental covariates included *season*, *habitat type* (hereafter just *habitat*), and *elevation*. We created the covariate *season* as a 2-level (summer and winter) categorical covariate. We used the covariates *habitat* and *elevation* as indicators of habitat characteristics. Habitat was a 4-level categorical covariate (broad-leaved forest, coniferous forest, mixed forest, and human land-use area), which we obtained from the CORINE Land Cover database (CLC 2012). We used the “spatial analyst” tool in ArcGIS 10.1 to cut the 100 × 100 m^2^ resolution vegetation layer according to coordinates of Turkey (36° to 42°N; 26° to 45°E); and with the “resample” function, we lowered the resolution to 2 × 2 km^2^ grid cells. We extracted the habitat-type category with the largest surface in each of the grid cells. Then, we assigned these categories to the camera-trap stations according to their coordinates using the R-package “raster” (Hijmans 2019). We calculated *elevation* as a continuous covariate from a digital elevation model (DEM), which we obtained from the 75 × 75 m^2^ GTOPO30 global elevation model (USGS-Earth Resources Observation and Science Center 1999) by using the “spatial analyst tool” in ArcGIS 10.1 and the R-package “raster.” We cropped the DEM according to coordinates of Turkey and lowered the resolution to 2 × 2 km^2^ grid cells with the “resample” function. We then calculated the mean elevation of each 2 × 2 km^2^ with the R-package “raster” and extracted the corresponding values for the analyses. Although lowering the spatial resolution of the covariates may reduce the model fit, resampling to 2 × 2 km^2^ was necessary to match the resolution of the covariates to the spacing of the camera traps. Finally, as anthropogenic covariate we used *rural human population density* (hereafter termed *human population*). We extracted the human population size from the Central Dissemination System Database of the Turkish Statistical Institute (TUIK 2012) using the “spatial analyst” tool in ArcGIS 10.1 and the R-package “raster.” We then calculated *human population*, a continuous variable, as population size per km^2^ by using the “point density” function. We used the “point density” function to exclude large cities and other urban areas and include only those areas that are associated with wilderness, such as villages and small towns. We standardized the continuous covariates by subtracting from each value the mean and dividing the difference by the standard deviation.

### Analysis framework and dynamic occupancy modeling.

We considered each camera-trap station as 1 sampling site. For each species per site, we created detection histories with 1 value per month: 1 if the species was detected, 0 if the species was not detected, and NA if the site was not sampled in a given month (e.g. during the 43-month sampling gap from October 2010 to April 2014, or due to camera malfunction or inaccessibility). We provide species records in Supplementary Data SD1 (Table S1). We structured the detection histories into 11 summer and 11 winter seasons, which we modeled as 22 primary sampling periods. The summer and winter seasons comprised 4 and 6 months, respectively, resulting in detection histories of 110 months. These 110 months were modeled as secondary (i.e. within season) sampling periods. In a given month, different camera traps may have worked for a different number of days (Supplementary Data SD2, Table S2). To account for the effect of this difference, we included the covariate *number of days* in the analysis of detection probability.

We used dynamic occupancy models to investigate how seasonality affects the habitat use of the study species. The framework of the dynamic occupancy model is similar to Pollock’s robust design (Pollock 1982), with secondary sampling periods within primary sampling periods (in our study, months within seasons). The occupancy state of a site remains unchanged between secondary sampling periods but may change between primary sampling periods. These changes are parameterized with the probabilities of local extinction and colonization (MacKenzie et al. 2006). In the context of our study, however, the terms “occupancy” and “extinction” need clarification. Because a camera-trap site may cover only a small portion of the home range of an individual, dynamic occupancy analysis of camera-trap data estimates probability of use of a site, rather than of occupancy, by the focal species (Mackenzie and Royle 2005). Hereafter, we therefore use the term “use,” instead of “occupancy.” Moreover, because disappearance from a site represents desertion rather than local extinction, we will refer to the probability of extinction as the probability of “desertion.” In our analysis, we used season-specific desertion and colonization probabilities to investigate changes in seasonal habitat use.

We characterized changes in the use of a site by a 2-state hierarchical model. One state describes changes in the ecological process, while the other state describes the observation process. We estimated 4 parameters from the model: the probability of site *i* being used in the first season (initial probability of use, ψ_*i*,1_), the probability of unused site *i* in season *t* to become used in season *t +* 1 (colonization probability, γ_*i*,*t*_), the probability of used site *i* in season *t* to become unused in season *t* + 1 (desertion probability, ε_*i*,*t*_), and the probability of detecting a species, given the species is present at site *i* during month *j* within season *t* (detection probability, *p*_*i*,*j*,*t*_). Based on these parameters, we estimated the probability of site *i* being used in primary sampling period *t* as a latent variable (true use state, *z*_*i,t*_) with a Bernoulli distribution of the sum of 2 probabilities: the probability that the site was used in the previous primary sampling period and the species did not desert the site plus the probability that the site was unused in the previous primary sampling period but was colonized since then (MacKenzie 2006; MacKenzie et al. 2006):


$${z_{i\text{,}t}}^{\sim }\text{ Bernoulli((}z_{i\text{,}t-\text{1}}*\text{(1}-\varepsilon_{i\text{,}t-\text{1}}\text{))}+\text{ ​​}\;\text{​​ ((1}-z_{i\text{,}t-\text{1}}\text{)}*\gamma_{i\text{,}t-\text{1}}\text{))}$$

To investigate potential seasonal fluctuations in the proportion of used sites, we summed the seasonal true use states over the sites. Additionally, in the observation model, we estimated the probability of detection with a Bernoulli distribution of the true use state, *z*_*i,t*_ multiplied with the estimated detection probability while accounting for the sampling effort, *p*_*i*,*j*,t_^*^. For the sampling effort, we used the number of days a camera trap was functioning at site *i* during primary sampling period *t*:


$$\begin{array}{l} {p_{i,j,t}}^{*}=1-{\left(1-p_{i,j,t}\right)}^{number\;of\;days} \end{array}$$

We applied the same 3 models, 1 additive model and 2 interaction models, with a logit-linear function to all species (Table 1). All models had the logistic regression component to estimate the latent true use state from the estimated colonization and desertion probabilities. In the additive model, regression models for colonization and desertion probabilities included the additive effects of the 3 covariates: *season*, *elevation*, and *human population* (Table 1). We included the latter 2 covariates because the sampling area had an elevation gradient and was under intensive human influence. Previous studies showed that these 2 factors can positively or negatively affect the distribution of different species in the area (Soyumert 2010). The regression model for detection probability included the interaction effect of *season* and *habitat*, because the habitat type affects detection and habitat accessibility may change seasonally. To estimate the latent true use state, the regression model of the initial probability of use included the interaction effect of *habitat* and *human population* to account for the use differences among habitat types under human influence. Finally, to account for potential differences among the 10 camera-trap areas (Fig. 1), all regression models included a random area effect. In the interaction models, we only changed the regression models for colonization and desertion probabilities (Table 1)—including the covariates *season* and either *human population* and the interaction between *season* and *human population* (interaction model I), or *elevation* and the interaction between *season* and *elevation* (interaction model II; Table 1).

**Table 1.** Additive and interaction models applied to all species. In each model, ψ is initial occupancy probability, γ is colonization probability, ε is desertion probability, and *p* is detection probability. The covariate *popden* is human population density, and *re(area)* is the random effect of area. Plus signs and colons indicate additive and interaction effects, respectively.

Model name	Model
Additive model	ψ ~ *habitat* + *popden* + *habitat*:*popden* + *re(area)* γ ~ *season* + *elevation* + *popden* + *re(area)* ε ~ *season* + *elevation* + *popden* + *re(area)* *p* ~ *season* + *habitat* + *season*:*habitat* + *re(area)*
Interaction model I: season & popden	ψ ~ *habitat* + *popden* + *habitat*:*popden* + *re(area)* γ ~ *season* + *popden* + *season*:*popden* + *re(area)* ε ~ *season* + *popden* + *season*:*popden* + *re(area)* *p* ~ *season* + *habitat* + *season*:*habitat* + *re(area)*
Interaction model II: season & elevation	ψ ~ *habitat* + *popden* + *habitat*:*popden* + *re(area)* γ ~ *season* + *elevation* + *season*:*elevation* + *re(area)* ε ~ *season* + *elevation* + *season*:*elevation* + *re(area)* *p* ~ *season* + *habitat* + *season*:*habitat* + *re(area)*

We applied a robust Bayesian framework using JAGS (Plummer 2003) and the R-package “jagsUI” (Kellner 2021) in R 4.0.2 (R Development Core Team 2022). We chose a Bayesian approach because it can account for uncertainty in the model output caused by temporally incomplete data. Additionally, in JAGS we have the flexibility to use weakly informative priors for multiple covariates in the same model and implement random effects more easily under Markov Chain Monte Carlo (MCMC) simulations. For each JAGS run, we used MCMC simulation settings under Gibbs sampling with regularizing priors: logistic distribution (dlogis(0, 1)) on intercepts, normal distribution (dnorm(0, 1)) on coefficients, and truncated normal distribution (dnorm(0, 1)T(0,)) on random effects. The MCMC settings comprised 100,000 adaptation iterations under 3 parallel chains and a thinning rate of 20 iterations, yielding a total of 135,000 samples for each model. All models converged with a 100,000-step burn-in phase, followed by 1,000,000 iterations. We checked convergence visually by diagnostic plots and the Brooks–Gelman–Rubin convergence diagnostic R-hat; R-hat ≤ 1.1 indicates convergence (Gelman and Rubin 1992). To check model fits, we applied goodness-of-fit (GoF) tests to the open (habitat-use transitions) and closed (detection) parts of each model. We calculated the Bayesian *P*-value based on chi-square (χ^2^) discrepancy for binomial data as Pr(χ^2^_replicated_ > χ^2^_observed_); values close to 0.5 indicate a good model fit, whereas values close to 0 or 1 suggest a poor model fit (Gelman 2013; Rankin et al. 2016). In addition, we calculated a “lack-of-fit” ratio as χ^2^_observed_/χ^2^_replicated_, which is expected to equal 1 if the model fits the data perfectly (Kéry and Schaub 2012). We also tested for significance of the covariates by checking on caterpillar plots whether posterior distributions of estimated intercepts and coefficient values overlap with zero. Because our aim was to test the effects of season, elevation, human population, and their interaction effects on changes in habitat-use patterns of a species, we focused on the estimated intercept and coefficient values of colonization and desertion probabilities (Supplementary Data SD3, Figs. S4 and S5). An example code for the Bayesian analysis is also provided in the Supplementary Data SD4.

## Results

The GoF test indicated a poor fit for most models on species detection probability and habitat-use transition (Supplementary Data SD3, Table S3). As indicated by the Bayesian *P*-values (Bpv), the lack of fit was greater for detection (the closed part of the models) than for habitat use (the open part of the models) in all species except the additive model from Eurasian Lynx (Bpv_closed_ = 0.14, Bpv_open_ = 0.09). In all models (additive, interaction model I, and interaction model II) the model fit was good for habitat use in Brown Bear (Bpv_open_ = 0.74) and Gray Wolf (Bpv_open_ = 0.76). For the other species, the Bpv_open_ were either close to 0 or 1, indicating a poor fit (Supplementary Data SD3, Fig. S1, Table S3). On the other hand, the lack-of-fit ratios indicated an acceptable model fit for habitat use, but a poor fit for detection (Supplementary Data SD3, Table S3). Additionally, according to the posterior distributions (Supplementary Data SD3, Figs. S4 and S5), the intercept and coefficient of the covariates *season*, *elevation*, and *human population* included in the models for colonization and desertion probabilities were significant, as were the interaction effect of *season* and *human population* in interaction model I and the interaction effect of *season* and *elevation* in interaction model II.

Detection probabilities did not differ significantly between seasons or among habitat types. In all species, they were close to 0 and, except in Eurasian Lynx, had narrow (<0.1) Bayesian credible intervals (BCIs). More detailed results are reported in the Supplementary Data SD6 (Fig. S10).

**Fig. 2.**
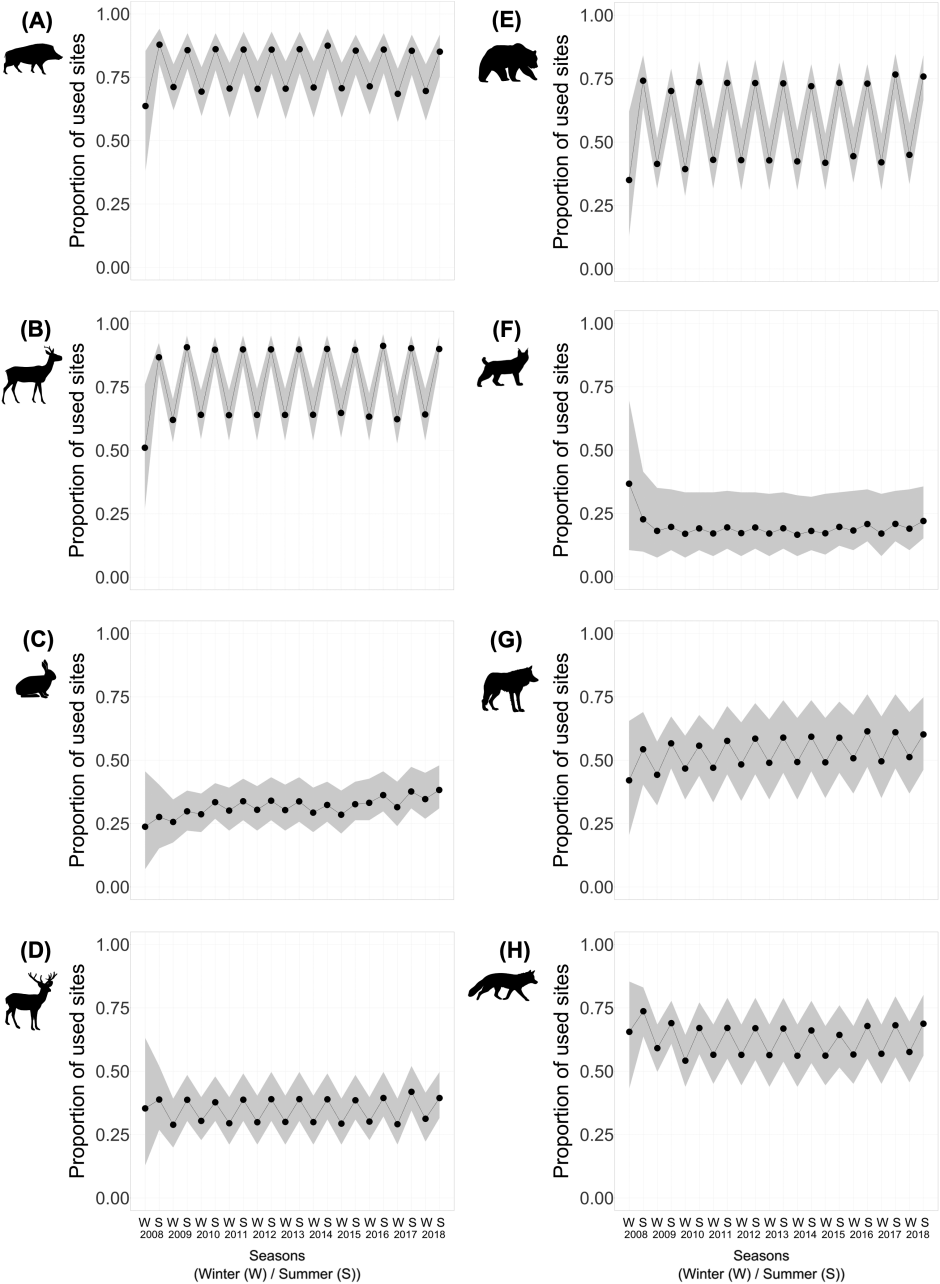
Seasonal proportion of sites used. The left column shows the prey species Wild Boar (A), Roe Deer (B), European Hare (C), and Red Deer (D); the right column shows the predator species Brown Bear (E), Eurasian Lynx (F), Gray Wolf (G), and Red Fox (H). Gray areas represent the 95% Bayesian credible intervals.

Since the GoF tests indicated no significant difference between the additive and interaction models (Supplementary Data SD3, Table S3), here, we show predictions from only the interaction models (predictions from the additive models can be found in Supplementary Data SD5, Figs. S8 and S9). According to these models, seasonality in habitat use occurred in all 8 species (Fig. 2). All species used more sites in summer than in winter, indicating seasonal range expansions and contractions. However, seasonality in habitat use was most pronounced in the Wild Boar, Roe Deer, and Brown Bear (Fig. 2A, B, and E), and least pronounced in the European Hare and Eurasian Lynx (Fig. 2C and F).

**Fig. 3.**
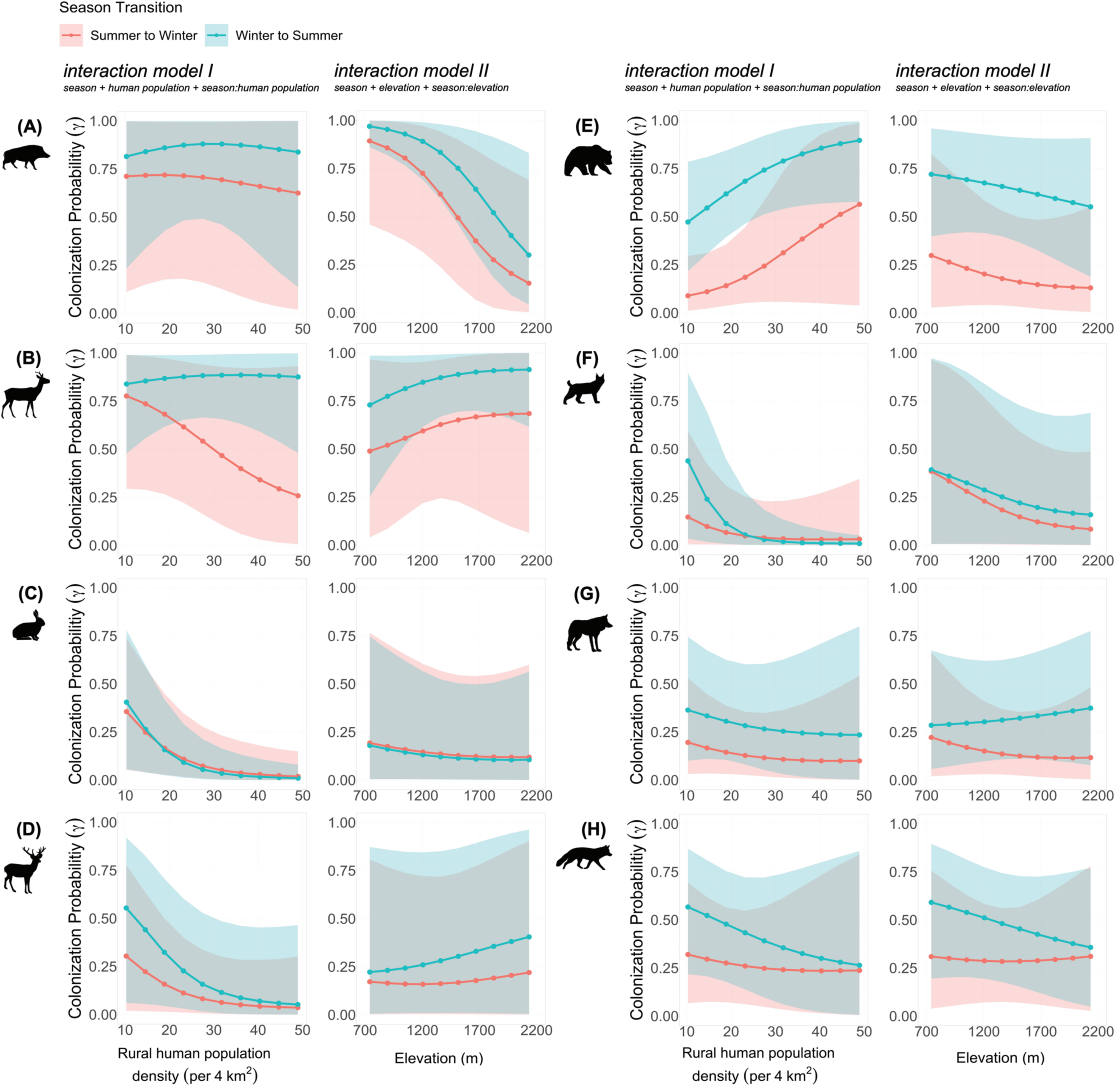
Seasonal colonization probabilities as a function of rural human population density (interaction model I) and elevation (interaction model II). The 2 columns on the left show the prey species Wild Boar (A), Roe Deer (B), European Hare (C), and Red Deer (D); the 2 columns on the right show the predator species Brown Bear (E), Eurasian Lynx (F), Gray Wolf (G), and Red Fox (H). Colored areas represent the 95% Bayesian credible intervals.

Seasonality in colonization probability was observed in 6 species—Wild Boar, Roe Deer, Red Deer, Brown Bear, Gray Wolf, and Red Fox (Fig. 3). In these species, colonization probability was always higher in winter-to-summer (W–S) than summer-to-winter (S–W) transitions. In line with results for habitat use, seasonality in colonization probability was most pronounced in Wild Boar, Roe Deer, and Brown Bear. Yet in all species, the 95% BCIs of the 2 transition periods were large and overlapped in both interaction models, indicating that the effect of season was weak. Colonization probability decreased at sites with higher human population in all but 1 species (Fig. 3, interaction model I), increasing in Brown Bear it increased. Elevation, on the other hand, affected the species differently (Fig. 3, interaction model II). With increasing elevation, the colonization probability decreased in both transition periods in Wild Boar, Brown Bear, and Eurasian Lynx (Fig. 3A, E and F), and increased in Roe Deer (Fig. 3B). In the other species, results differed for the 2 transition periods. In Red Deer and Gray Wolf, colonization probability slightly increased with increasing elevation in W–S transitions but remained unchanged in S–W transitions (Fig. 3D and G), whereas in Red Fox, it decreased with increasing elevation in W–S transitions but remained unchanged in S–W transitions (Fig. 3H). In European Hare, colonization probability remained unchanged along the elevation gradient in both transition periods (Fig. 3C).

**Fig. 4.**
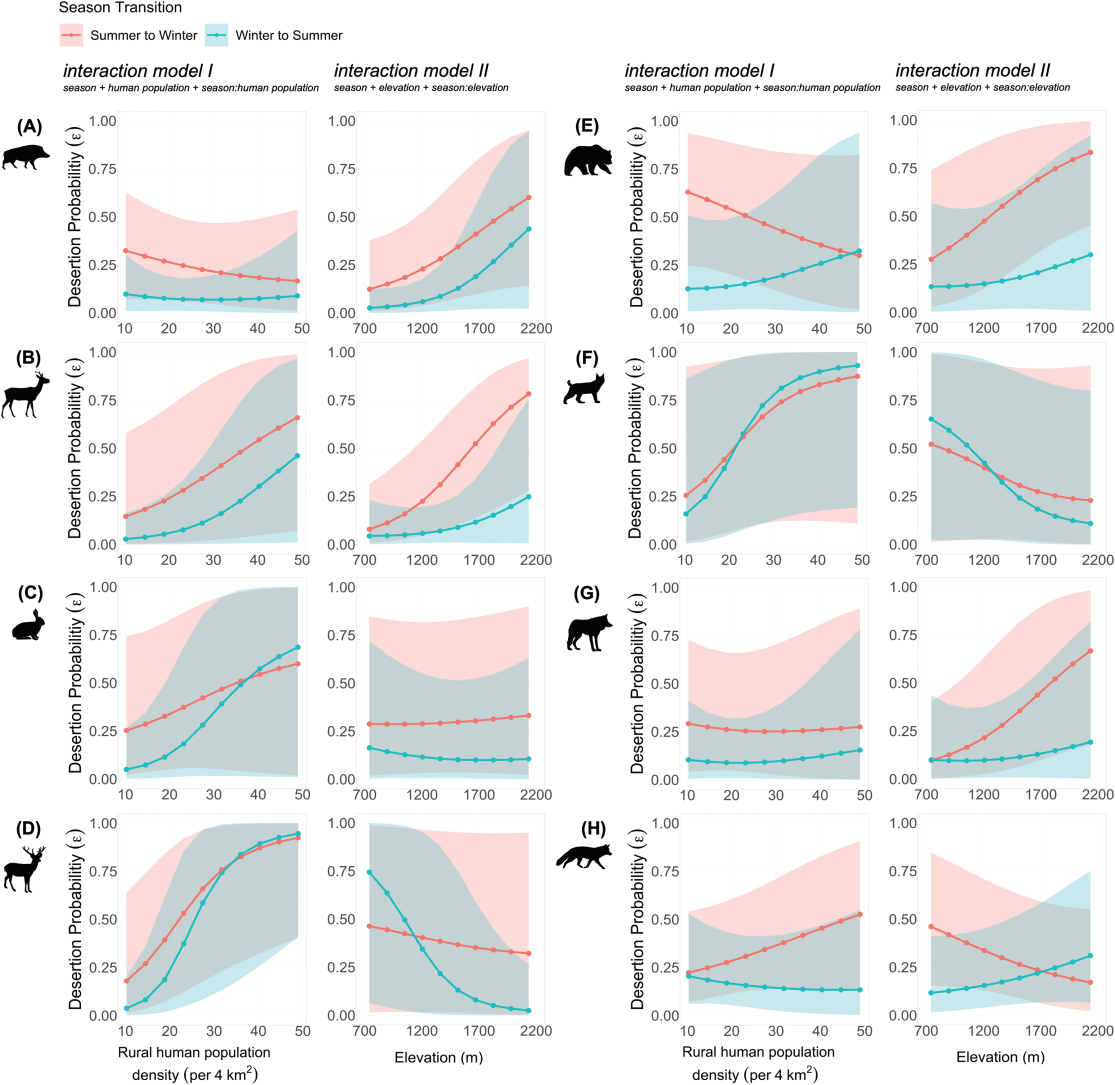
Seasonal desertion probabilities as a function of rural human population density (interaction model I) and elevation (interaction model II). The 2 columns on the left show the prey species Wild Boar (A), Roe Deer (B), European Hare (C), and Red Deer (D); the 2 columns on the right show the predator species Brown Bear (E), Eurasian Lynx (F), Gray Wolf (G), and Red Fox (H). Colored areas represent the 95% Bayesian credible intervals.

Seasonality in desertion probability was observed in all species (Fig. 4). Yet again, the 95% BCIs were large and overlapped in both interaction models, indicating that the effect of season was weak. At sites with lower human population, desertion probabilities were always higher in S–W than W–S transitions (Fig. 4, interaction model I). Moreover, with increasing human population, the desertion probability increased in both transition periods in Roe Deer, European Hare, Red Deer, and Eurasian Lynx (Fig. 4B, C, D, and F), and remained unchanged in Gray Wolf (Fig. 4G). In the other species, the results differed for the 2 transition periods. In Wild Boar, desertion probability decreased in S–W transition and remained unchanged in W–S transition (Fig. 4A); in Brown Bear, it increased in W–S transition and decreased in S–W transition (Fig. 4E); and in Red Fox, it increased in S–W transition and remained unchanged in W–S transition (Fig. 4H).

## Discussion

We used dynamic occupancy models to identify seasonal habitat-use patterns of 8 large-mammal species in a spatially and temporally heterogeneous, human-dominated landscape, and studied how elevation and human population affected these patterns. All species used a higher proportion of sites in summer than in winter. We found particularly strong seasonality in habitat use in Wild Boar, Roe Deer, and Brown Bear. Below, we discuss these findings and highlight potential mechanisms underlying the observed variation in the strength of seasonality among species (Lacher et al. 2019).

In our study area, seasonality in a species habitat use could result from seasonality in at least 3 factors—a species resource availability and abundance, breeding phenology, and human activities and associated disturbances, as described below.

First, resources fluctuate both temporally and spatially (Stein et al. 2014). In our study area, food resources are generally more widespread and accessible during summer (Soyumert 2010). For herbivores and omnivores like Wild Boar, Roe Deer, and Brown Bear, fluctuating food resources include seasonal crops, fruits, seeds, and plants (Clevenger et al. 1990; Morellet et al. 2013; Morelle and Lejeune 2015; Ambarli et al. 2016). For strict carnivores like Gray Wolf, Lynx, and Red Fox, they include seasonally varying prey density and availability (Fuller and Sievert 2001; Macdonald and Reynolds 2004). As a result of these fluctuations, species can be more widespread in summer. In winter, on the other hand, snow cover and snowmelt can restrict access to food and shelter (Slatyer et al. 2021). Because food is scarce in winter and energetic costs of moving through snow are high, species may avoid sites with a high snow cover (e.g. Nelson and Mech 1986; Mysterud et al. 1997).

Second, annual breeding cycle and related activities of a species can lead to fluctuations in local abundance and habitat use (Nielsen et al. 2010). For example, observed increases in habitat use of Roe Deer, Red Deer, and Gray Wolf in summer might be caused by the search for mating partners (Jayakody et al. 2008; Mech and Boitani 2010; Ertürk 2017; Hagen et al. 2017). Breeding activity also correlates with food availability, because many species time breeding when food is abundant (Taylor et al. 2013).

Third, habitat use of the study species may also be affected by seasonality in human activities in the region. During winter, human activities are concentrated around cities and towns, leaving rural areas mostly undisturbed. In summer, people return to their farmlands. As a result, most human activities in rural areas take place during summer, such as agriculture and forestry. The increase in human activity in summer may also increase hunting pressure, and heavily hunted species such as Wild Boar, Roe Deer, Red Deer, and European Hare may be more widely distributed in order to avoid sites with a high human activity (Soyumert 2010). European Hare and Red Deer are restricted to the eastern part of the study area (Soyumert 2010). Increasing human pressure in this region may restrict the distribution of these 2 species further, thus weakening seasonality in habitat use even more (Smith et al. 2004; Jayakody et al. 2008; Coppes et al. 2017).

Several other factors might also cause, or obscure, seasonality in habitat use. For example, predators like Gray Wolf and Eurasian Lynx roam large areas, which may obscure seasonality effects in their habitat use (Breitenmoser 2000; Mancinelli et al. 2018). Moreover, in social species like Gray Wolf, social behavior can also affect seasonal activity. For example, pack formation and changes in social pressure within packs (e.g. when pack size increases, the amount of food per individual decreases) may lead to increased activity during summer and explain the seasonality observed in their habitat use (Metz et al. 2011). Lastly, seasonal territoriality of some species (e.g. Roe Deer, Red Deer, Gray Wolf, and Eurasian Lynx) can also explain their seasonal habitat-use patterns and distribution (Carranza et al. 1990; Schmidt et al. 1997; Rossi et al. 2003; Mech and Boitani 2010).

Seasonality was less pronounced in colonization probability than in desertion probability. As expected, because food is more widespread and abundant in summer (Soyumert 2010), colonization probability was higher in winter-to-summer than summer-to-winter transitions in all species. In the case of Brown Bear, its inactivity period likely contributed to this pattern (Linnell et al. 2000). Desertion probability, on the other hand, was higher in summer-to-winter than winter-to-summer transitions, indicating that the species deserted certain sites (e.g. where food is scarce) in search of suitable overwintering places. In several species, however, seasonal differences in desertion probability were affected by elevation and human population. In European Hare, Red Deer, and Eurasian Lynx, for example, desertion probability was higher in winter-to-summer than summer-to-winter transitions at low-elevation sites and sites with a high human population. These reversed seasonality patterns are likely related to human avoidance (Chapman and Flux 1990; Jayakody et al. 2008; Filla et al. 2017). As mentioned before, human settlements in the study area are mostly at low elevations, and the human population increases in summer. However, uncertainties in the estimates of colonization and desertion probabilities were large. More data from the ongoing survey and covariates with better spatiotemporal resolution will hopefully reduce the uncertainties and lead to clearer results.

As expected, colonization probability decreased with increasing human population in all but 1 species, Brown Bear, who may colonize sites that are close to human settlements in search of food and den sites, which could explain the observed trend (Linnell et al. 2000; Treves and Karanth 2003). The other species appear to avoid humans, possibly because they are hunted or poached as they are either popular game species (e.g. Roe Deer, European Hare, and Red Deer; Chapman and Flux 1990; Benhaiem et al. 2008; Jayakody et al. 2008; Coppes et al. 2017) or threats to livestock and poultry (e.g. Gray Wolf, Red Fox, and Eurasian Lynx; Breitenmoser 2000; Macdonald and Reynolds 2004; Albayrak 2011; Ertürk 2017).

Counteracting the trend for colonization probability, desertion probability increased with increasing human population in Roe Deer, European Hare, Red Deer, and Eurasian Lynx in both seasonal transitions. These trends might result from increasing human activity in summer, underlining the negative effect of human disturbance on habitat-use patterns. The same pattern was also observed in Red Fox, but only during summer-to-winter transitions—because human population and activity are low in winter, this pattern may be related to the availability of the main prey, small rodents, which are more widespread toward the end of summer and the beginning of winter (Macdonald and Reynolds 2004; Soyumert 2010). In Wild Boar and Gray Wolf, however, desertion probability was overall low and did not change with human population density. For these species, food and prey availability might be a more dominant driver in seasonal habitat use than human disturbance (Mech and Boitani 2010; Thurfjell et al. 2013). In Brown Bear, on the other hand, desertion probability decreased with increasing human population in summer-to-winter transitions but slightly increased in winter-to-summer transitions. The latter trend is expected, because the species avoids sites with a high human population density during summer (Clevenger et al. 1990—the former trend may result from it foraging for anthropogenic food sources (e.g. garbage) during food shortage in winter (Ambarli 2016) and its preference for den sites in dense forests (Linnell et al. 2000). Even though such sites may be close to human settlements, dense forests provide better shelter, especially during their inactivity period (Linnell et al. 2000).

Elevation correlates with food availability and human pressure and affects habitat use of large mammals. In our study area, low-elevation sites have denser forests and thus provide more food and shelter than high-elevation sites, but also have higher human population densities. Based on the more food and better shelter argument, habitat use can be expected to increase with decreasing elevation. However, based on the higher human population argument, habitat use can be expected to decrease with decreasing elevation. Moreover, because food availability and human population vary seasonally, these expectations might depend on season, which would affect expectations for colonization probability. Increasing elevation positively affected the colonization probability of Roe Deer and Red Deer. This result is in line with avoidance of humans by both species due to hunting pressure and preference of Red Deer for open areas at high-elevation sites (Benhaiem et al. 2008; Jayakody et al. 2008). On the other hand, increasing elevation negatively affected the colonization probability of Wild Boar, Brown Bear, Eurasian Lynx, and Red Fox. For Wild Boar and Red Fox, this negative effect was expected, because these species highly prefer food sources near human settlements (e.g. agricultural crops for the Wild Boar and poultry for Red Fox; Macdonald and Reynolds 2004; Smith et al. 2004; Morelle and Lejeune 2015). For the Brown Bear and Eurasian Lynx, dense forests at low-elevation sites provide better shelter (Linnell et al. 2000; Filla et al. 2017). In addition, Eurasian Lynx may also prefer low-elevation sites because of increased prey availability, but it also avoids humans by moving to higher elevations. Our results suggest that the lynx prioritizes prey availability over human avoidance (Filla et al. 2017).

Effects of elevation on desertion probability were in line with effects of elevation on colonization probability in some but not all species. Increasing elevation positively affected the desertion probability of Wild Boar and Brown Bear, and negatively affected the desertion probability of Red Deer. The positive trend of Wild Boar might again result from preference to forage near human settlements, whereas the positive trend of Brown Bear might result from better shelter in dense forests at lower elevations. For Red Deer, the negative trend may result from human avoidance and preference to forage in open areas (Jayakody et al. 2008). For remaining species, however, effects of elevation on desertion and colonization probabilities showed the same trend in both parameters. For example, in Roe Deer and Gray Wolf, desertion probability increased with increasing elevation. Roe Deer might prioritize feeding habits over human avoidance and prefer more productive deciduous forests at low-elevation sites closer to humans (Benhaiem et al. 2008). In Gray Wolf, the trend might result from indirect effects of elevation on their prey species (Mech and Boitani 2010). In both Roe Deer and Gray Wolf, increasing snow cover with increasing elevation might also contribute to the positive effect of elevation on desertion probability during summer-to-winter transitions. Because snow cover negatively affects resource availability and movement, both species may desert sites that become covered in snow (Mech and Boitani 2010; Morellet et al. 2013).

In conclusion, we showed that seasonality affects habitat use of large-mammal species in a human-dominated landscape. However, the patterns and strength of seasonality varied substantially among species. These variances were caused by differences in life history and their response to environmental and anthropogenic factors (i.e. resource availability, snow, elevation, and human pressure). These results indicate that environmental seasonality and its varying effects on seasonal habitat use are important factors to consider when designing a management plan for the region.

## Supplementary data

Supplementary data are available at *Journal of Mammalogy* online.

**Supplementary Data SD1.**—Species records.

**Supplementary Data SD2.**—Camera-trap activity.

**Supplementary Data SD3.**—Goodness-of-fit tests and posterior parameter distributions.

**Supplementary Data SD4.**—JAGS code for the dynamic occupancy model.

**Supplementary Data SD5.**—Predictions from additive models.

**Supplementary Data SD6.**—Detection probability of large mammals.

gyad107_suppl_Supplementary_Datas_SD1_Tables_S1

gyad107_suppl_Supplementary_Datas_SD2_Tables_S2

gyad107_suppl_Supplementary_Datas_SD3_Tables_S3_Figures_S1-S7

gyad107_suppl_Supplementary_Datas_SD4

gyad107_suppl_Supplementary_Datas_SD5_Figures_S8-S9

gyad107_suppl_Supplementary_Datas_SD6_Figures_S10

## Data Availability

All codes, including the Bayesian analysis, diagnostics, and the GoF test, are available on GitHub and can be accessed via https://github.com/dilsaddt/NorthAnatolia_2022_singlesp_seasonal_habitat_use.git.
