# Supplementary material for: Seasonal habitat-use patterns of large mammals in a human-dominated landscape
Source: J Mammal. 2023 Nov 24;105(1):122–33. doi: 10.1093/jmammal/gyad107 (PMC11275454; doi:10.1093/jmammal/gyad107)
Supplement: gyad107_suppl_Supplementary_Datas_SD1_Tables_S1 [file gyad107_suppl_supplementary_datas_sd1_tables_s1.docx]

**Title: Seasonal habitat-use patterns of large mammals in a human-dominated landscape**

Dilsad Dagtekin^a^ (ORCID ID: 0000-0001-8610-0835), Alper Ertürk^b^ (ORCID ID: 0000-0001-5498-3856), Stefan Sommer^a^ (ORCID ID: 0000-0002-4092-7068), Arpat Ozgul^a^ (ORCID ID: 0000-0001-7477-2642), Anil Soyumert^b^ (ORCID ID: 0000-0003-0196-9617)

^a^ Department of Evolutionary Biology and Environmental Studies, University of Zurich, Winterthurerstrasse 190, CH-8057 Zurich, Switzerland

^b^ Hunting and Wildlife Program, Araç Rafet Vergili Vocational School of Higher Education, Kastamonu University, TR-37800, Arac, Kastamonu, Turkey

Corresponding author: Dilsad Dagtekin - dilsad.dagtekin@ieu.uzh.ch

**Supporting Information SD1:** Species records.

**Table S1.** Species records.

| **Species** | **Presence (1)** | **Absence (0)** |
| --- | --- | --- |
| Wild boar (*Sus scrofa*) | 868 | 1956 |
| Roe deer (*Capreolus capreolus*) | 1175 | 1651 |
| European hare (*Lepus europaeus*) | 725 | 2096 |
| Red deer (*Cervus elaphus*) | 445 | 2377 |
| Brown bear (*Ursus arctos*) | 615 | 2207 |
| Eurasian lynx (*Lynx lynx*) | 208 | 2614 |
| Gray wolf (*Canis lupus*) | 600 | 2221 |
| Red fox (*Vulpes vulpes*) | 929 | 1893 |
