# Supplementary material for: Seasonal habitat-use patterns of large mammals in a human-dominated landscape
Source: J Mammal. 2023 Nov 24;105(1):122–33. doi: 10.1093/jmammal/gyad107 (PMC11275454; doi:10.1093/jmammal/gyad107)
Supplement: gyad107_suppl_Supplementary_Datas_SD3_Tables_S3_Figures_S1-S7 [file gyad107_suppl_supplementary_datas_sd3_tables_s3_figures_s1-s7.docx]

**Title: Seasonal habitat-use patterns of large mammals in a human-dominated landscape**

Dilsad Dagtekin^a^ (ORCID ID: 0000-0001-8610-0835), Alper Ertürk^b^ (ORCID ID: 0000-0001-5498-3856), Stefan Sommer^a^ (ORCID ID: 0000-0002-4092-7068), Arpat Ozgul^a^ (ORCID ID: 0000-0001-7477-2642), Anil Soyumert^b^ (ORCID ID: 0000-0003-0196-9617)

^a^ Department of Evolutionary Biology and Environmental Studies, University of Zurich, Winterthurerstrasse 190, CH-8057 Zurich, Switzerland

^b^ Hunting and Wildlife Program, Araç Rafet Vergili Vocational School of Higher Education, Kastamonu University, TR-37800, Arac, Kastamonu, Turkey

Corresponding author: Dilsad Dagtekin - dilsad.dagtekin@ieu.uzh.ch

**Supporting Information SD3:** Goodness-of-fit test and posterior parameter distributions.

We conducted  goodness-of-fit tests based on posterior predictive distributions for the open (habitat-use transitions) and closed (detection probability) parts of  all additive and interaction models for each species (Gelman, 2013). We used and adapted the code from Rankin et al. (2016) and used chi-square (χ^2^) statistics to calculate Bayesian p-values (Bpv) and lack-of-fit statistics (Kéry and Schaub 2012). We followed these steps for 37,500 MCMC samples for both observed and replicate datasets: For the open part of each model, we (1) simulated replicate detection and true habitat-use values, (2) calculated the four possible transitions in a habitat with estimated colonization and desertion probabilities, (3) computed expected numbers of transitions, (4) computed the χ^2^ value from the expected number of transitions, and (5) summed up the χ^2^ values over all transitions and seasons. For the closed part of each model, we computed the detection frequencies for each site and season and calculated the χ^2^ value (Kéry and Royle, 2021). We then calculated the Bpv from the proportion of samples where the discrepancy of the replicated data was greater than the discrepancy of the observed data  (χ^2^_replicated_ > χ^2^_observed_) (Figs. S1 and S2). A Bpv close to either 0 or 1 indicates a poor model fit (Gelman, 2013). We also calculated a ‘lack-of-fit’ ratio as χ^2^_observed_ / χ^2^_replicated_. If a model fits the data perfectly, this ratio should be 1 (Kery & Schaub, 2012).

|  |  | ***Additive model*** | | ***Interaction model I*** | | ***Interaction model II*** | |
| --- | --- | --- | --- | --- | --- | --- | --- |
| **Species** | **Model part** | **Bayesian p-value** | **Lack-of-fit ratio** | **Bayesian p-value** | **Lack-of-fit ratio** | **Bayesian p-value** | **Lack-of-fit ratio** |
| Wild boar  (*Sus scrofa)* | Open part | 0.95 | 0.990 | 0.93 | 0.991 | 0.94 | 0.990 |
|  | Closed part | 0.00 | 1.680 | 0.00 | 1.662 | 0.00 | 1.675 |
| Roe deer  (*Capreolus capreolus)* | Open part | 0.99 | 0.989 | 0.99 | 0.998 | 0.99 | 0.989 |
|  | Closed part | 0.00 | 2.019 | 0.00 | 2.008 | 0.00 | 2.012 |
| European hare  (*Lepus europaeus)* | Open part | 0.04 | 1.006 | 0.05 | 1.006 | 0.04 | 1.006 |
|  | Closed part | 0.00 | 2.350 | 0.00 | 2.305 | 0.01 | 2.947 |
| Red deer  (*Cervus elaphus)* | Open part | 0.02 | 1.010 | 0.02 | 1.010 | 0.03 | 1.008 |
|  | Closed part | 0.00 | 2.076 | 0.00 | 2.081 | 0.01 | 2.330 |
| Brown bear  (*Ursus arctos)* | Open part | 0.74 | 0.997 | 0.81 | 0.996 | 0.72 | 0.998 |
|  | Closed part | 0.00 | 1.727 | 0.00 | 1.732 | 0.00 | 1.723 |
| Eurasian lynx  (*Lynx lynx)* | Open part | 0.09 | 1.008 | 0.14 | 1.006 | 0.15 | 1.004 |
|  | Closed part | 0.14 | 5.429 | 0.09 | 4.793 | 0.36 | 6.825 |
| Gray wolf  (*Canis lupus)* | Open part | 0.76 | 0.998 | 0.75 | 0.997 | 0.75 | 0.998 |
|  | Closed part | 0.00 | 1.640 | 0.00 | 1.651 | 0.00 | 1.652 |
| Red fox  (*Vulpes vulpes)* | Open part | 0.90 | 0.997 | 0.89 | 0.997 | 0.92 | 0.996 |
|  | Closed part | 0.00 | 2.040 | 0.00 | 2.028 | 0.00 | 2.023 |

**Table S3.**Bayesian p-values and lack-of-fit ratios calculated from goodness-of-fit tests for the additive and the interaction models. Open and closed parts of the models indicate habitat-use transition and detection probability, respectively.


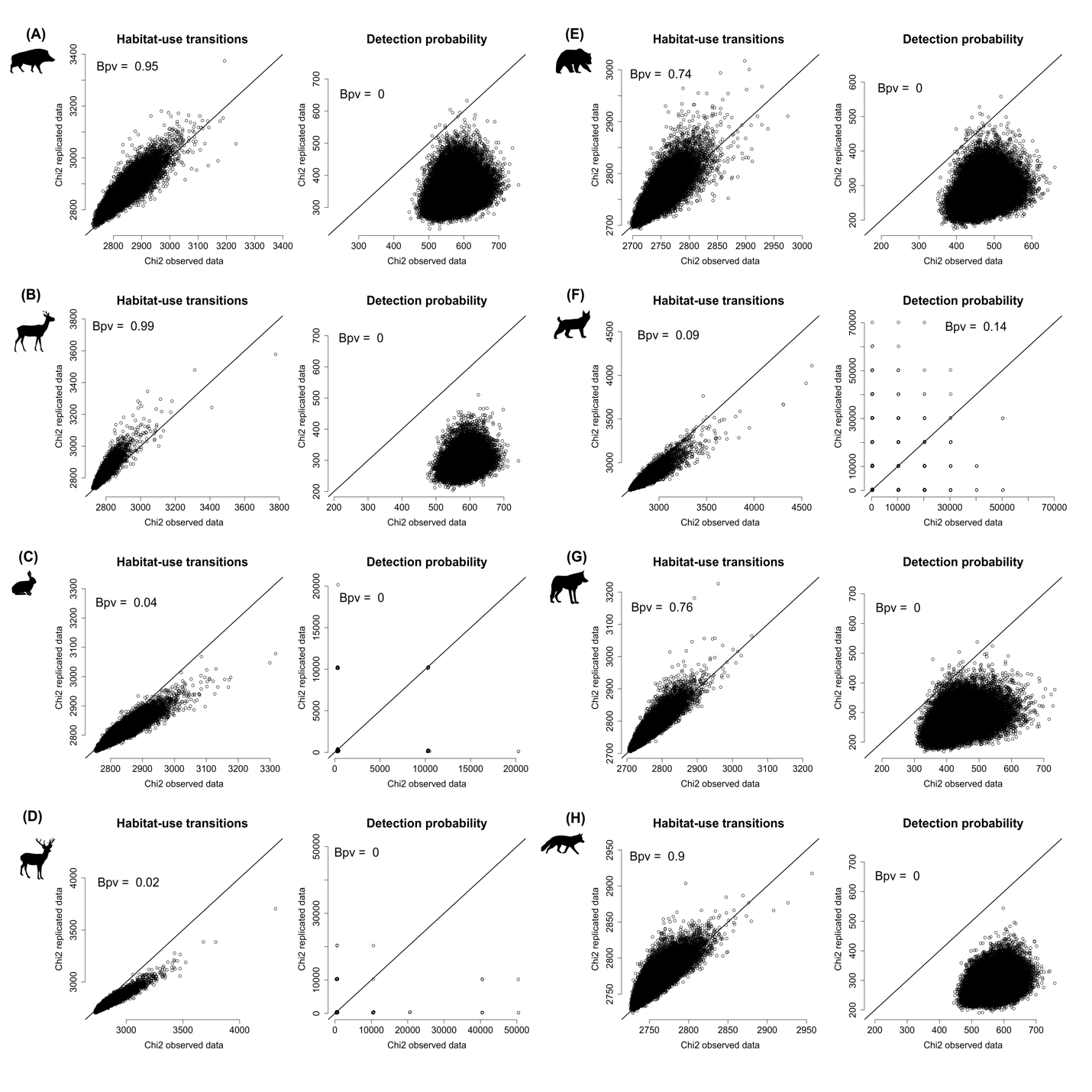


**Fig. S1.** Species-specific goodness-of-fit tests for habitat-use transition (open part of the models) and detection probability (closed part of the models) from the additive models. The Bayesian p-value (Bpv) is the proportion of points above the x = y diagonal.


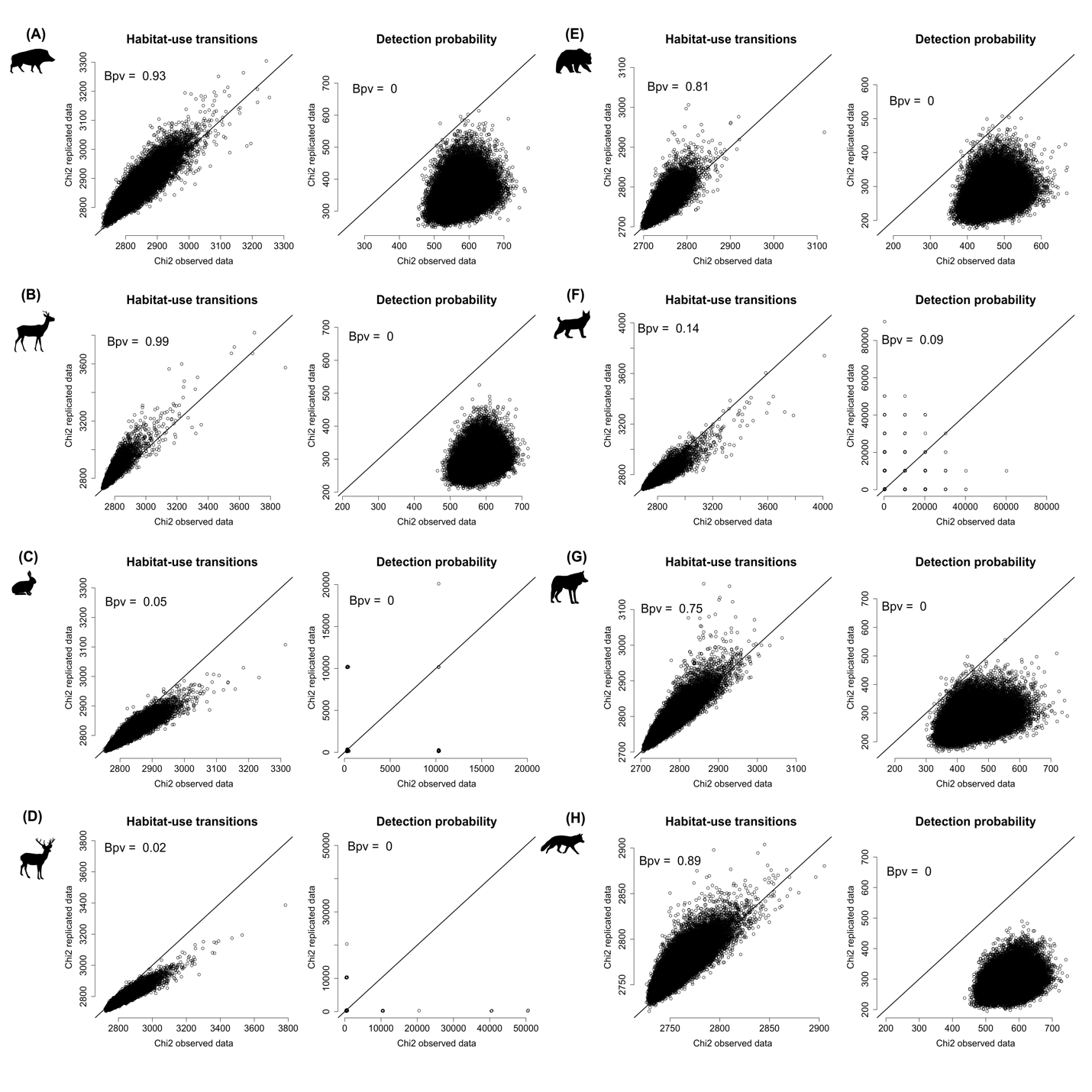


**Fig. S2.** Species-specific goodness-of-fit tests for habitat-use transition (open part of the models) and detection probability (closed part of the models) from the interaction models I. The Bayesian p-value (Bpv) is the proportion of points above the x = y diagonal.


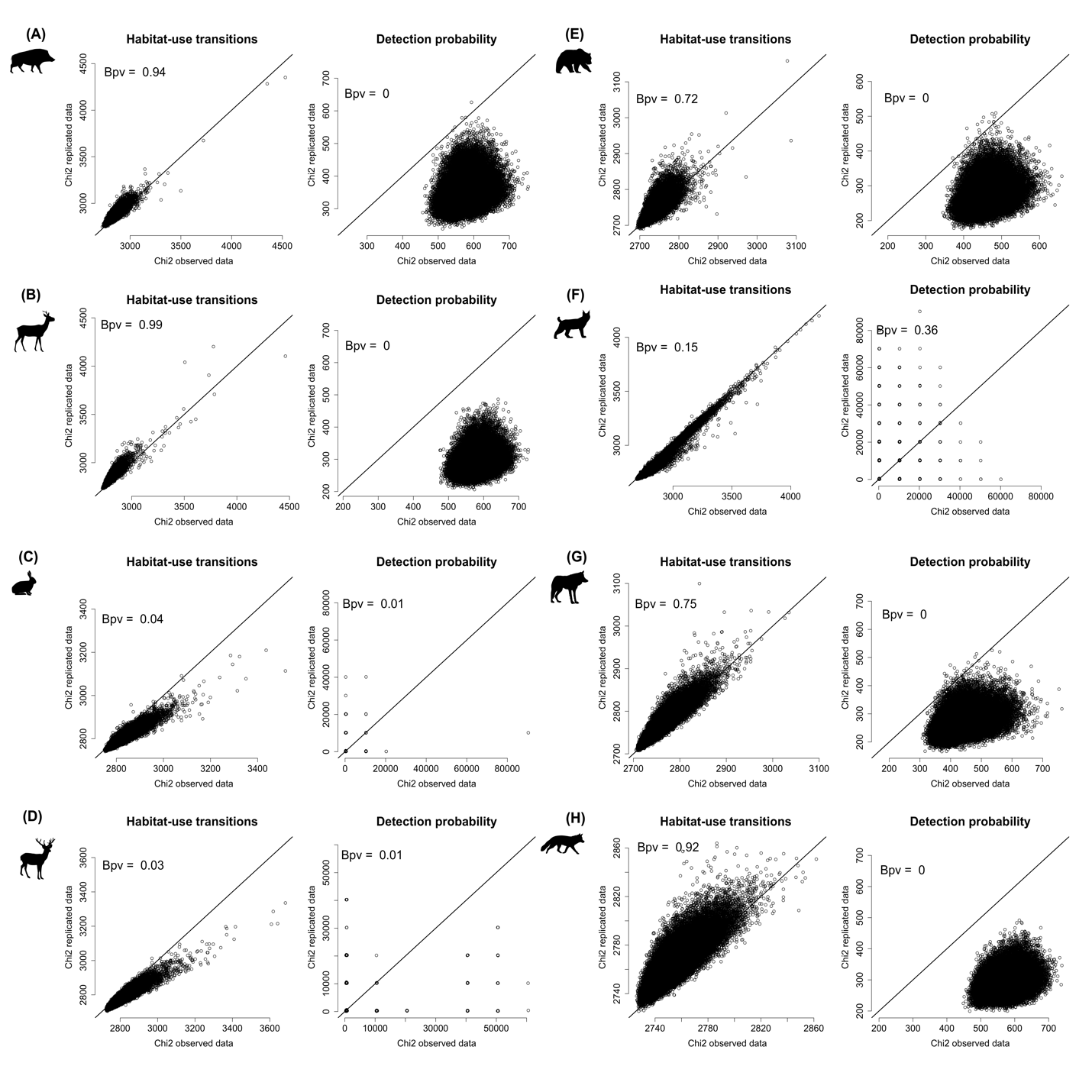


**Fig. S3.** Species-specific goodness-of-fit tests for habitat-use transition (open part of the models) and detection probability (closed part of the models) from the interaction models II. The Bayesian p-value (Bpv) is the proportion of points above the x = y diagonal.

We tested for significance of the covariates by visually checking on caterpillar plots whether posterior distributions of estimated intercepts and coefficient values overlap with zero (Figs. S4 and S5). Because colonization and desertion parameters were indicators of changes in species’ habitat-use patterns, we focused on the estimated intercept and coefficient values of these parameters under the effects of season, elevation, and human population for the additive models; season, human population, and their interaction for the interaction models I; and season, elevation and their interaction for the interaction models II. We found that the effect of season, elevation, and human population on the colonization and desertion parameters of all species’ additive models were mainly significant. Almost all species had a few parameters that overlapped with zero in their credible intervals; however, the mean values did not overlap with zero (Fig. S4). The interaction effect of season and human population, and of season and elevation, on the colonization and desertion parameters were also significant in the majority of the species (Fig. S5). We also provided the caterpillar plots for the random area effect for each parameter (Figs. S6 and S7). These effects differed among the areas, especially for the detection probability; yet, they were weakly informative because their 95% Bayesian credible intervals overlapped with zero in all species' additive and interaction models.


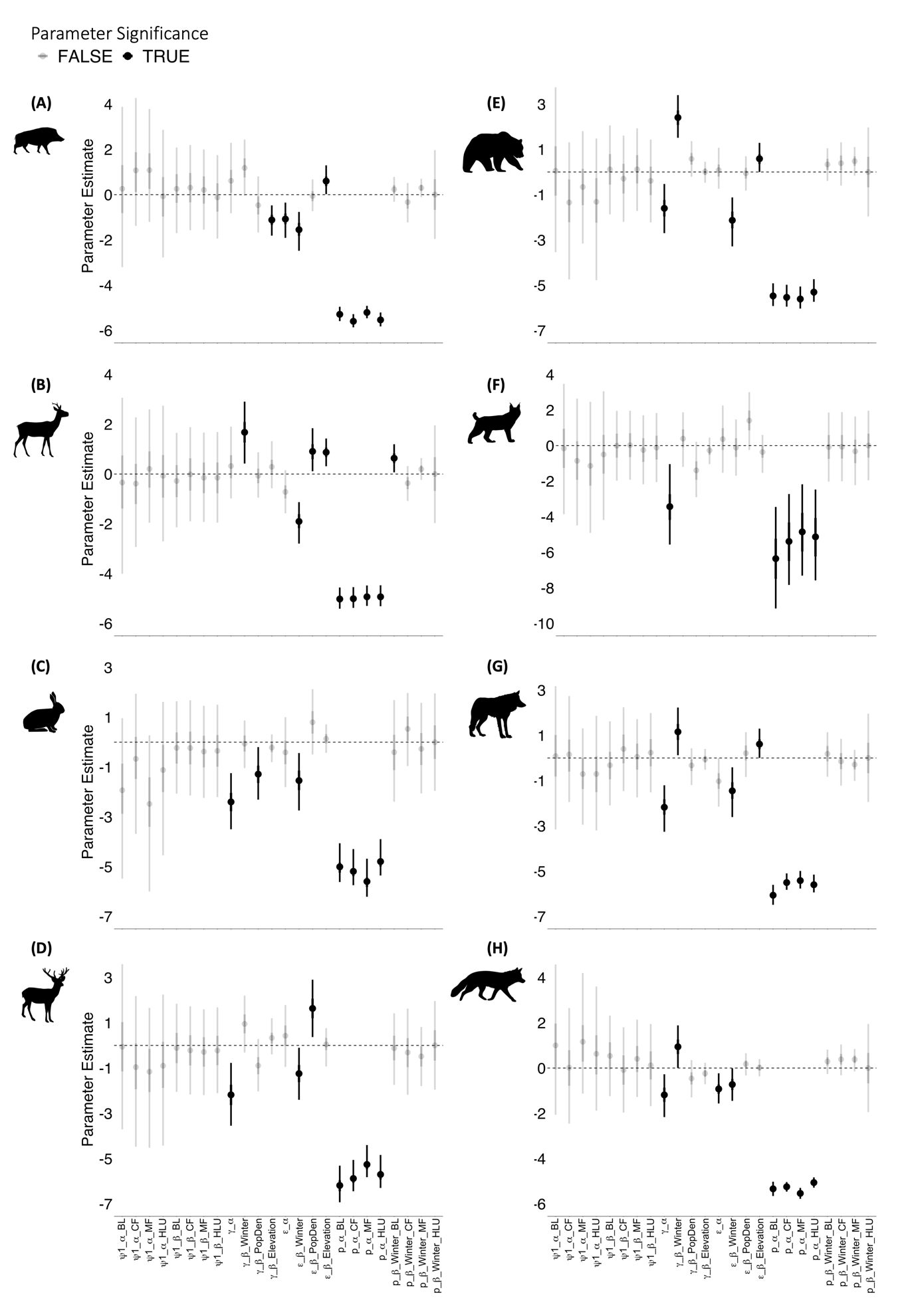


**Fig. S4.** Species-specific posterior parameter distributions from additive models. The covariates are *habitat type* (BL: broad-leaved forest, CF: coniferous forest, MF: mixed forest, HLU: human land-use area), *season* (Winter), *rural human population density* (popden), and *elevation*. Points represent posterior medians, thick lines represent 50% Bayesian credible intervals, and thin lines represent 95% Bayesian credible intervals. If the 95% Bayesian credible interval of a given parameter includes zero, that parameter is considered statistically non-significant (shown in gray). Parameters are labeled as follows: initial probability of use, $\psi_{1}$; colonization probability, $\gamma$, desertion probability, $\varepsilon$; and detection probability, $p$. Intercepts, coefficients, and random effects are labeled $\alpha$ and $\beta$, respectively.


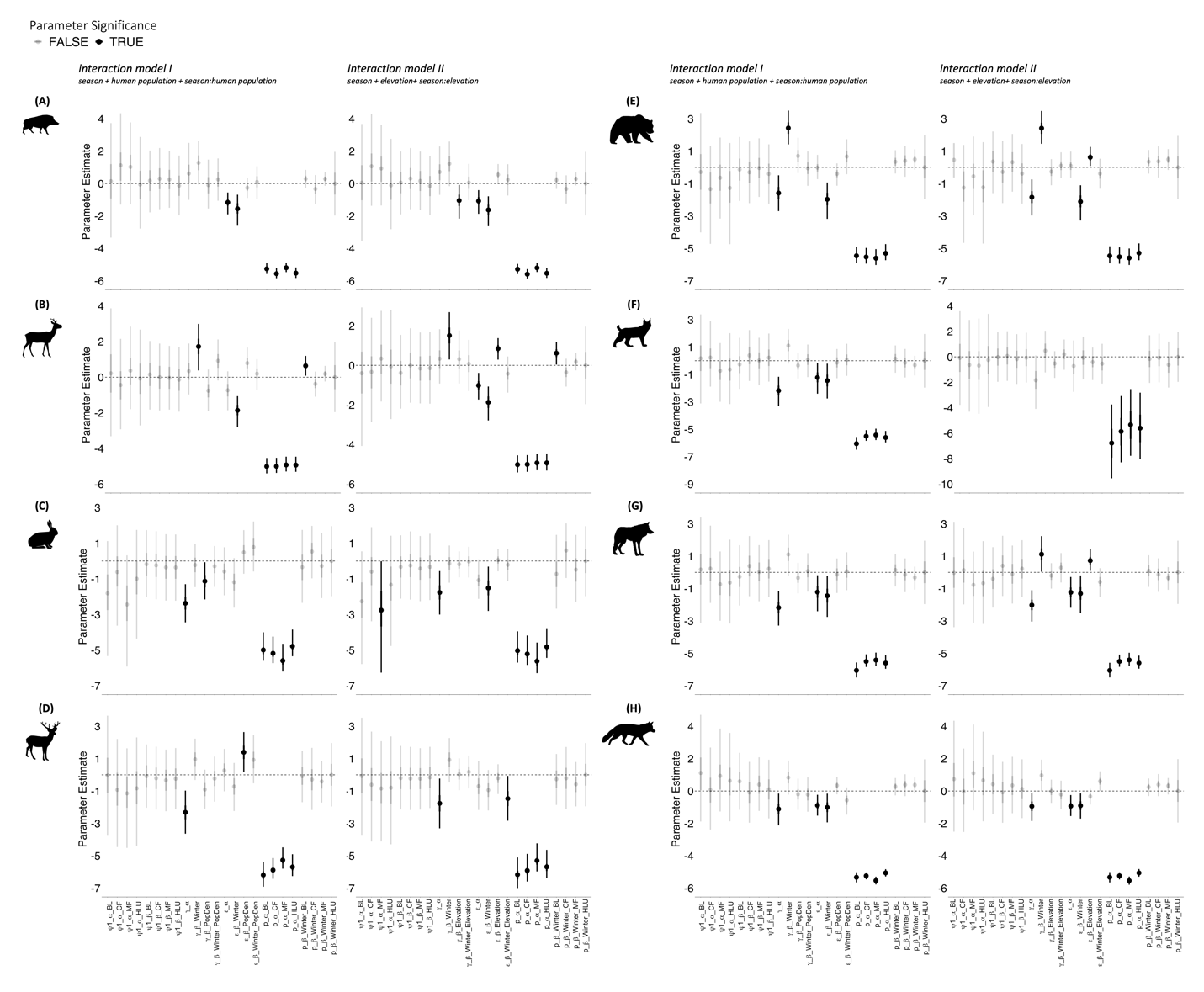


**Fig. S5.** Species-specific posterior parameter distributions from interaction models I and II. The covariates are *habitat type* (BL: broad-leaved forest, CF: coniferous forest, MF: mixed forest, HLU: human land-use area), *season* (Winter), *rural human population density* (popden), and *elevation*. Points represent posterior medians, thick lines represent 50% Bayesian credible intervals, and thin lines represent 95% Bayesian credible intervals. If the 95% Bayesian credible interval of a given parameter includes zero, that parameter is considered statistically non-significant (shown in gray). Parameters are labeled as follows: initial probability of use, $\psi_{1}$; colonization probability, $\gamma$, desertion probability, $\varepsilon$; and detection probability, $p$. Intercepts, coefficients, and random effects are labeled $\alpha$ and $\beta$, respectively.


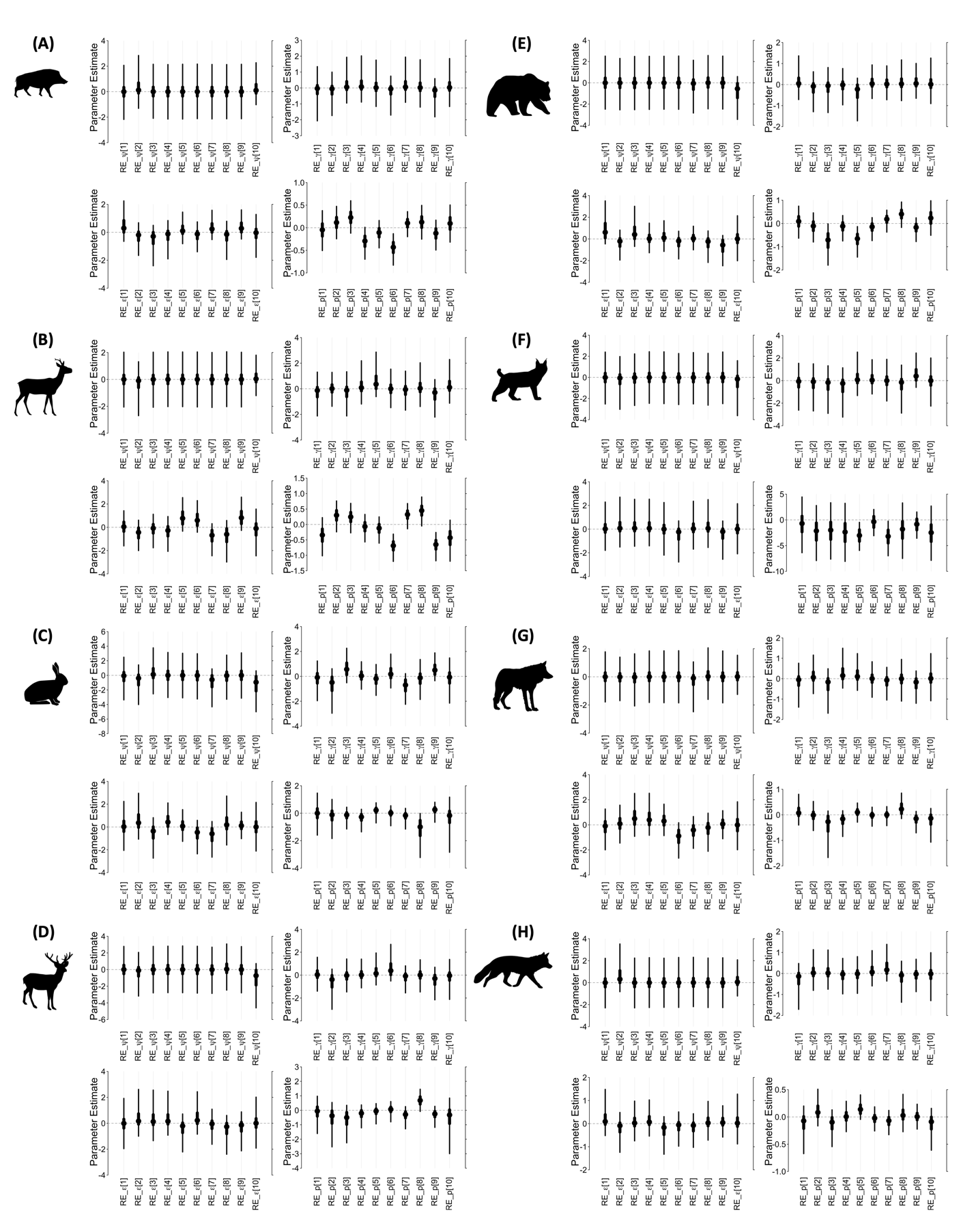


**Fig. S6.** Species-specific posterior parameter distributions for random area effects (RE) from additive models. Points represent posterior medians, thick lines represent 50% Bayesian credible intervals, and thin lines represent 95% Bayesian credible intervals. If the 95% Bayesian credible interval of a given parameter includes zero, that parameter is considered statistically non-significant. Parameters are labeled as follows: initial probability of use, $\psi_{1}$; colonization probability, $\gamma$, desertion probability, $\varepsilon$; and detection probability, $p$.


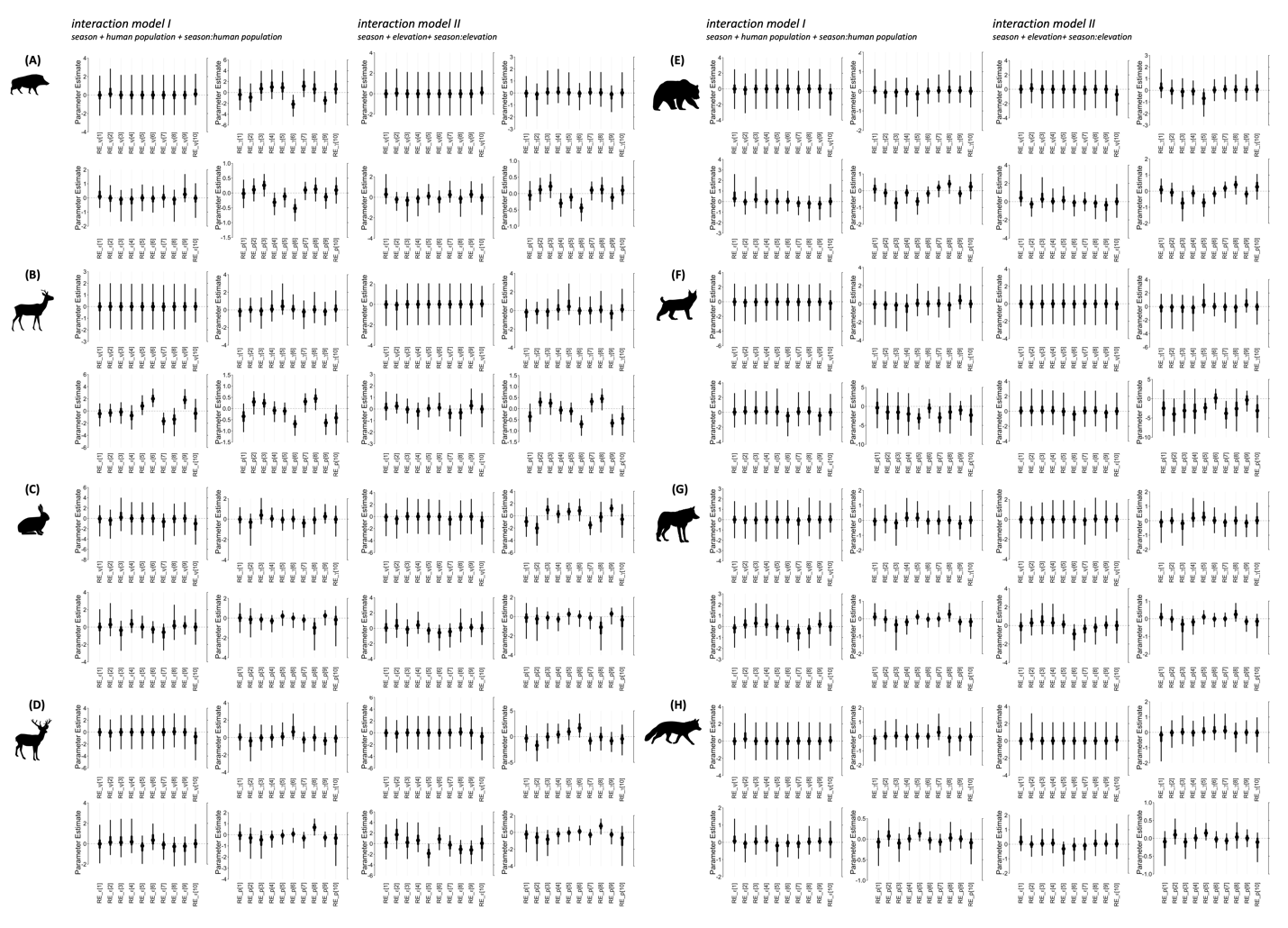


**Fig. S7. S**pecies-specific posterior parameter distributions for random area effects (RE) from interaction models I and II. Points represent posterior medians, thick lines represent 50% Bayesian credible intervals, and thin lines represent 95% Bayesian credible intervals. If the 95% Bayesian credible interval of a given parameter includes zero, that parameter is considered statistically non-significant. Parameters are labeled as follows: initial probability of use, $\psi_{1}$; colonization probability, $\gamma$, desertion probability, $\varepsilon$; and detection probability, $p$.

## References

Gelman, A. (2013). Two simple examples for understanding posterior p-values whose distributions are far from uniform. *Electronic Journal of Statistics*, 7, 2595-2602. https://doi.org/10.1214/13-EJS854

Kéry, M., & Royle, J. A. (2021). Applied Hierarchical Modeling in Ecology: Analysis of distribution, abundance and species richness in R and BUGS: Volume 2: Dynamic and Advanced Models. Academic Press.

Kéry, M., & Schaub, M. (2012). Bayesian population analysis using WinBUGS: a hierarchical perspective. Academic Press.

Rankin, R. W., Nicholson, K. E., Allen, S. J., Krützen, M., Bejder, L., & Pollock, K. H. (2016). A full-capture Hierarchical Bayesian model of Pollock's Closed Robust Design and application to dolphins*. Frontiers in Marine Science*, 3, 25. https://doi.org/10.3389/fmars.2016.00025
