# Supplementary material for: Seasonal habitat-use patterns of large mammals in a human-dominated landscape
Source: J Mammal. 2023 Nov 24;105(1):122–33. doi: 10.1093/jmammal/gyad107 (PMC11275454; doi:10.1093/jmammal/gyad107)
Supplement: gyad107_suppl_Supplementary_Datas_SD4 [file gyad107_suppl_supplementary_datas_sd4.docx]

**Title: Seasonal habitat-use patterns of large mammals in a human-dominated landscape**

Dilsad Dagtekin^a^ (ORCID ID: 0000-0001-8610-0835), Alper Ertürk^b^ (ORCID ID: 0000-0001-5498-3856), Stefan Sommer^a^ (ORCID ID: 0000-0002-4092-7068), Arpat Ozgul^a^ (ORCID ID: 0000-0001-7477-2642), Anil Soyumert^b^ (ORCID ID: 0000-0003-0196-9617)

^a^ Department of Evolutionary Biology and Environmental Studies, University of Zurich, Winterthurerstrasse 190, CH-8057 Zurich, Switzerland

^b^ Hunting and Wildlife Program, Araç Rafet Vergili Vocational School of Higher Education, Kastamonu University, TR-37800, Arac, Kastamonu, Turkey

Corresponding author: Dilsad Dagtekin - [dilsad.dagtekin@ieu.uzh.ch](mailto:dilsad.dagtekin@ieu.uzh.ch)

**Supporting Information SD4:** JAGS code for the dynamic occupancy model.

library(jagsUI)

 # Specify model in BUGS language

cat(file = "cc_112022_full_alphap.txt","

model {

# Model: cc_112022_full_alphap

#

# psi1 ~ habitat + popden + habitat:popden + re.psi1(area)

# gamma ~ season + popden + elevation + re.gammma(area)

# eps ~ season + popden + elevation + re.eps(area)

# p ~ season + habitat + season:habitat + re.p(area)

# -----------------------------------------------------------------------------------------

#

# Parameters:

#

# psi1: initial occupancy probability

# gamma: colonization probability

# eps (epsilon) : desertion probability

# p: detection probability

# -----------------------------------------------------------------------------------------

#

# Others:

#

# narea: number of study areas, 10

# nsite: number of camera-trap stations, 171

# nprimary: total number of primary sampling periods, seasons, 22

# nobs: number of observations

# y: detection history

# z: true latent state variable, z=1 means occupied, z=0 not occupied.

# n.occ: number of occupied sites

# n.prop: proportion of occupied sites to all total sites

#

# area: study areas

# season: seasons, 2-level categorical, winter(1) and summer (0)

# popden: rural human population density, continuous, standardized

# elevation: meter, continuous, standardized

# habitat: habitat type, 4-level categorical:

## Broad Leaved Forest (BL) = 1

## Coniferous Forest (CF) = 2

## Mixed Forest (MF) = 3

## Human Land-Use Areas (O) = 4

#

# -----------------------------------------------------------------------------------------

#

#### PRIORS ####

# psi1 ~ habitat + popden + habitat:popden + re.psi1(area)

for (h in 1:4) {

  alphapsi[h] ~ dlogis(0, 1)                                    # intercept for each habitat type(4)

  # betap values should be according to habitat types (AHM II Chp15 pp.104-105)

  # we have 4 habitat types --> 4 betapsi values for 1 betapsi category

  # we have 1 betapsi category: popden effect

  betapsi[h] ~ dnorm(0, 1)                                      # prior for popden coefficient w/ habitat types

}

# gamma ~ season + popden + elevation + re.gamma(area)

alphagamma ~ dlogis(0, 1)                                       # intercept

for(i in 1:3){                                                  # prior for coefficients

  betagamma[i] ~ dnorm(0, 1)

}

# eps ~ season + popden + elevation + re.eps(area)

alphaeps ~ dlogis(0, 1)                                         # intercept

for(i in 1:3){                                                  # prior for coefficients

  betaeps[i] ~ dnorm(0, 1)

}

# p ~ season*habitat

for (h in 1:4) {

  # alphap[h] ~ dlogis(0, 1)                                      # intercept for each habitat type(4)

  alphap[h] ~ dlogis(-2, 1)

  # betap values should be according to habitat types (AHM II Chp15 pp.104-105)

  # we have 4 habitat types --> 4 betap values for 1 betap category

  # we have 1 betap category: betap[p] season effect

  betap[h] ~ dnorm(0, 1)                                        # betap season effect

}

# Random effect priors

for (t in 1:(narea)){

randompsi[t] ~ dnorm(0, taupsi)

randomgamma[t] ~ dnorm(0, taugamma)

randomeps[t] ~ dnorm(0, taueps)

randomp[t] ~ dnorm(0, taup)

}

sigmapsi ~ dnorm(0, 1)T(0,)                    # Priors for standard deviations

sigmagamma ~ dnorm(0, 1)T(0,)

sigmaeps ~ dnorm(0, 1)T(0,)

sigmap ~ dnorm(0, 1)T(0,)

taupsi <- pow(sigmapsi, -2)

taugamma <- pow(sigmagamma, -2)

taueps <- pow(sigmaeps, -2)

taup <- pow(sigmap, -2)

sigma2psi <- pow(sigmapsi, 2)                  # Temporal variances

sigma2gamma <- pow(sigmagamma, 2)

sigma2eps <- pow(sigmaeps, 2)

sigma2p <- pow(sigmap, 2)

#### MODELS ####

# Ecological submodel: intial occupancy as derived parameter

for (i in 1:nsite){

  z[i,1] ~ dbern(psi1[i])

  logit(psi1[i]) <- alphapsi[habitat[i]]

  + betapsi[habitat[i]]*popden[i]

  + randompsi[area[i]]

}

# State transitions: colonization and extinction

# for col and ext

for (i in 1:nsite){

  for (s in 2:nprimary){

    logit(gamma[i,s-1]) <- alphagamma

    + betagamma[1]*season[i,s-1] + betagamma[2]*popden[i] + betagamma[3]*elevation[i]

    + randomgamma[area[i]]

    logit(eps[i,s-1]) <- alphaeps

    + betaeps[1]*season[i,s-1] + betaeps[2]*popden[i] + betaeps[3]*elevation[i]

    + randomeps[area[i]]

  }

}

# for z: true occupancy

for(i in 1:nsite){

  for (t in 2:nprimary){

    z[i,t] ~ dbern((z[i,t-1]*(1-eps[i,t-1])) + ((1-z[i,t-1])*gamma[i,t-1]))

  }

}

# Observation model

for (i in 1:nobs){

  logit(p[i]) <- alphap[habitat.p[i]]

  + betap[habitat.p[i]]*season.p[site[i]]

  + randomp[area.p[i]]

   #y[i] ~ dbern(z[site[i],pocc[i]]*p[i])  # y: observed occupancy

   pstar[i] <- 1-(1-p[i])^ndays[i]

   y[i] ~ dbern(z[site[i],pocc[i]]*pstar[i])  # y: observed occupancy

}

#### DERIVED PARAMETERS ####

# Compute population and sample occupancy

n.occ[1] <- sum(z[1:nsite,1])  # Number of occupied sites in sample

n.prop[1] <- n.occ[1]/nsite

for (t in 2:nprimary){

  n.occ[t] <- sum(z[1:nsite,t])

  n.prop[t] <- n.occ[t]/nsite

}

}")

# Initial values

inits<-function(){list(z=matrix(1,171,22))}

# Parameters monitored

params <- c("alphapsi", "betapsi",

            "alphagamma", "betagamma",

            "alphaeps", "betaeps",

            "alphap", "betap",

            "randompsi", "randomgamma", "randomeps", "randomp",

            "n.occ","n.prop")

# MCMC settings

na <- 100000

nb <- 100000

nt <- 20

ni <- 1000000

nc <- 3

parallel:::setDefaultClusterOptions(setup_strategy = "sequential")

# call JAGS

cc_112022_full_alphap <- jags(jags.data, inits, params, "cc_112022_full_alphap.txt", n.chains = nc, n.adapt = na, n.iter = ni, n.burnin = nb, n.thin = nt, parallel = TRUE)

print(cc_112022_full_alphap, 3)
