# Supplementary material for: Seasonal habitat-use patterns of large mammals in a human-dominated landscape
Source: J Mammal. 2023 Nov 24;105(1):122–33. doi: 10.1093/jmammal/gyad107 (PMC11275454; doi:10.1093/jmammal/gyad107)
Supplement: gyad107_suppl_Supplementary_Datas_SD5_Figures_S8-S9 [file gyad107_suppl_supplementary_datas_sd5_figures_s8-s9.docx]

**Title: Seasonal habitat-use patterns of large mammals in a human-dominated landscape**

Dilsad Dagtekin^a^ (ORCID ID: 0000-0001-8610-0835), Alper Ertürk^b^ (ORCID ID: 0000-0001-5498-3856), Stefan Sommer^a^ (ORCID ID: 0000-0002-4092-7068), Arpat Ozgul^a^ (ORCID ID: 0000-0001-7477-2642), Anil Soyumert^b^ (ORCID ID: 0000-0003-0196-9617)

^a^ Department of Evolutionary Biology and Environmental Studies, University of Zurich, Winterthurerstrasse 190, CH-8057 Zurich, Switzerland

^b^ Hunting and Wildlife Program, Araç Rafet Vergili Vocational School of Higher Education, Kastamonu University, TR-37800, Arac, Kastamonu, Turkey

Corresponding author: Dilsad Dagtekin - dilsad.dagtekin@ieu.uzh.ch

**Supporting Information SD5:** Predictions from additive models.


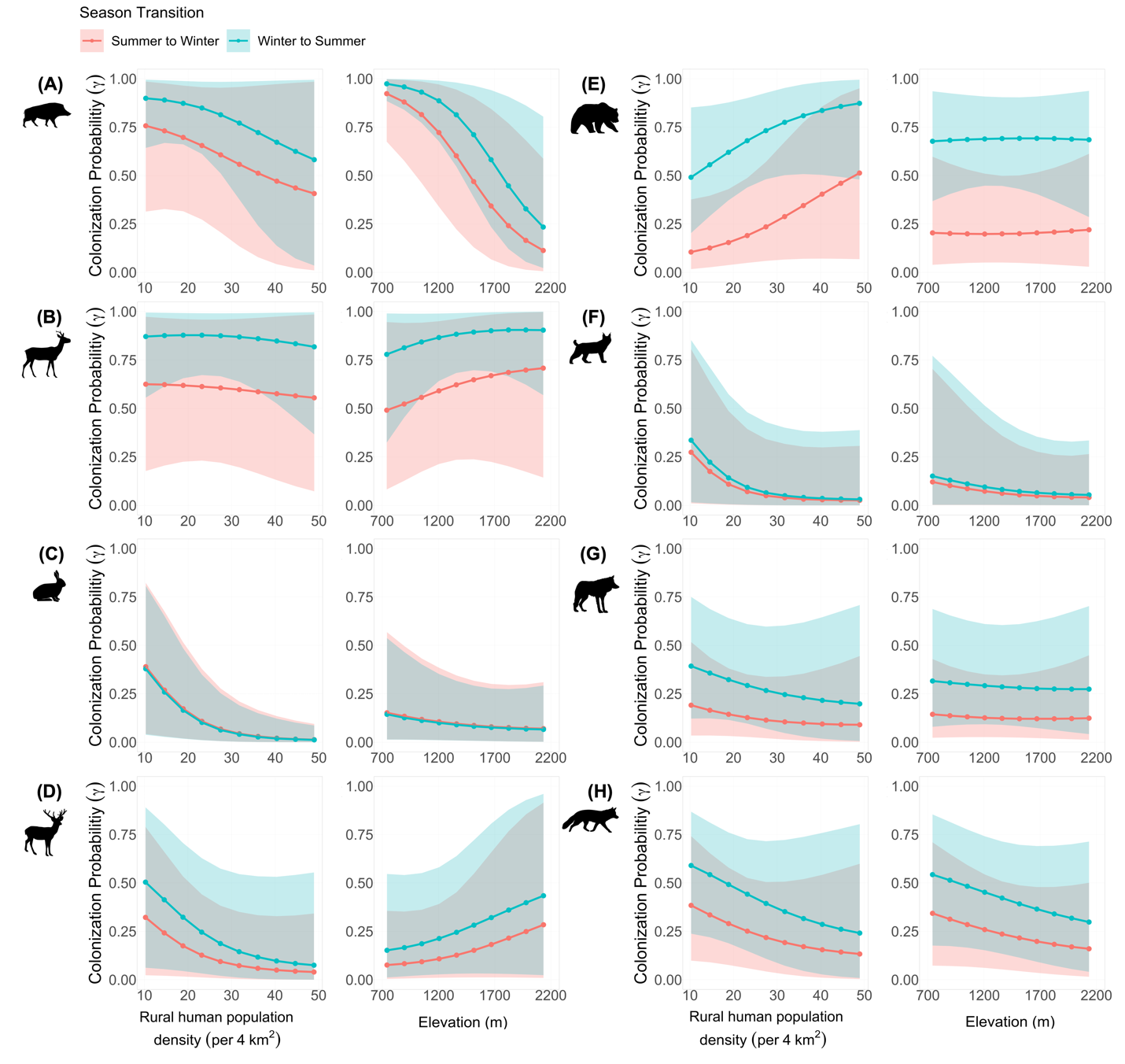


**Fig. S8.** Seasonal colonization probabilities as a function of season, rural human population density, and elevation (additive models). The two columns on the left show the prey species wild boar (A), roe deer (B), European hare (C), and red deer (D); the two columns on the right show the predator species brown bear (E), Eurasian lynx (F), gray wolf (G), and red fox (H). Colored areas represent the 95% Bayesian credible intervals.


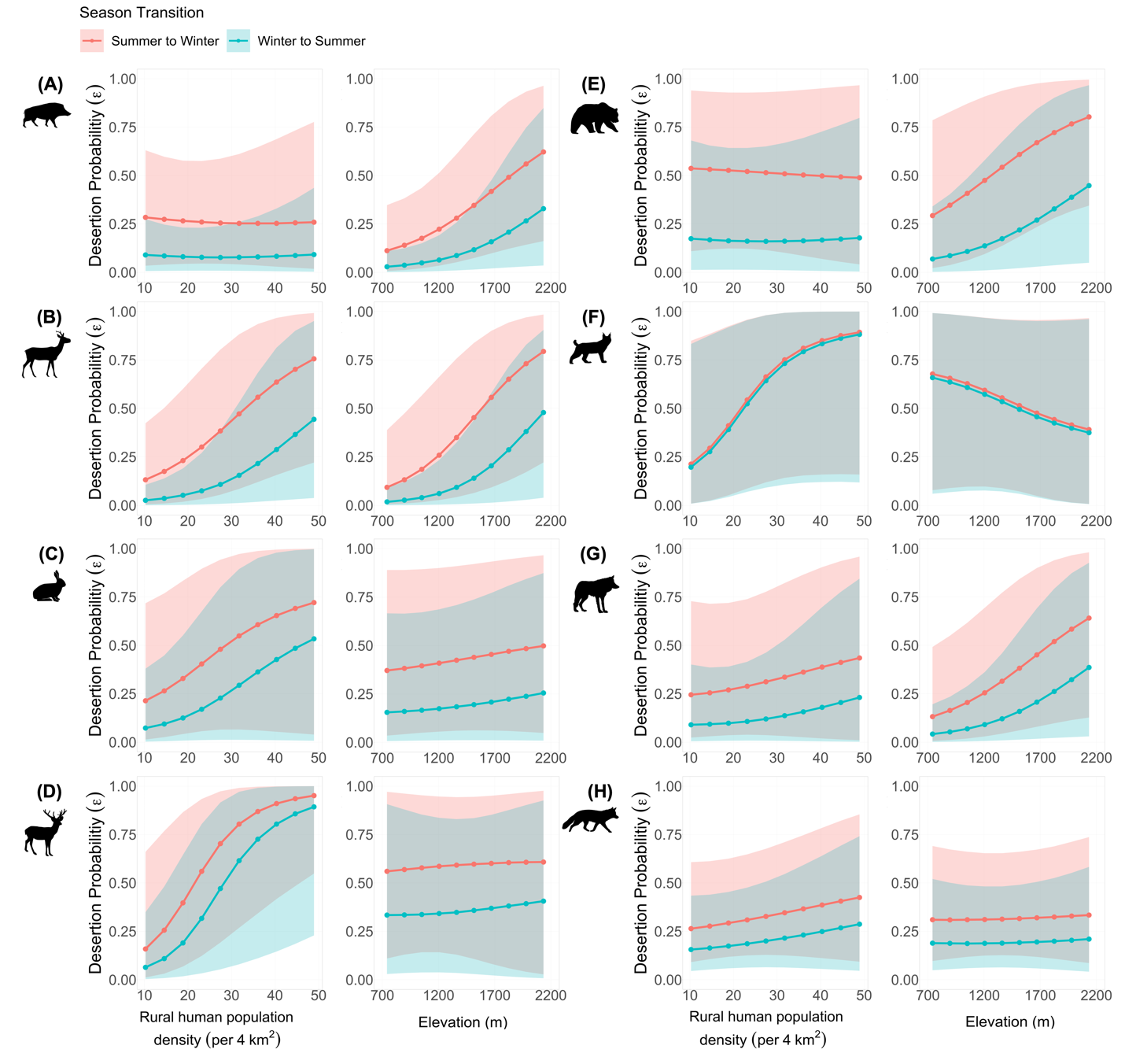


**Fig. S9.** Seasonal desertion probabilities as a function of season, rural human population density, and elevation (additive models). The two columns on the left show the prey species wild boar (A), roe deer (B), European hare (C), and red deer (D); the two columns on the right show the predator species brown bear (E), Eurasian lynx (F), gray wolf (G), and red fox (H). Colored areas represent the 95% Bayesian credible intervals.
