# Supplementary material for: Seasonal habitat-use patterns of large mammals in a human-dominated landscape
Source: J Mammal. 2023 Nov 24;105(1):122–33. doi: 10.1093/jmammal/gyad107 (PMC11275454; doi:10.1093/jmammal/gyad107)
Supplement: gyad107_suppl_Supplementary_Datas_SD6_Figures_S10 [file gyad107_suppl_supplementary_datas_sd6_figures_s10.docx]

**Title: Seasonal habitat-use patterns of large mammals in a human-dominated landscape**

Dilsad Dagtekin^a^ (ORCID ID: 0000-0001-8610-0835), Alper Ertürk^b^ (ORCID ID: 0000-0001-5498-3856), Stefan Sommer^a^ (ORCID ID: 0000-0002-4092-7068), Arpat Ozgul^a^ (ORCID ID: 0000-0001-7477-2642), Anil Soyumert^b^ (ORCID ID: 0000-0003-0196-9617)

^a^ Department of Evolutionary Biology and Environmental Studies, University of Zurich, Winterthurerstrasse 190, CH-8057 Zurich, Switzerland

^b^ Hunting and Wildlife Program, Araç Rafet Vergili Vocational School of Higher Education, Kastamonu University, TR-37800, Arac, Kastamonu, Turkey

Corresponding author: Dilsad Dagtekin - dilsad.dagtekin@ieu.uzh.ch

**Supporting Information SD5:** Detection probability of large mammals.

We tested the interaction effect of season and habitat type on the detection probability of the eight mammal species (Fig. S10). Because the area is a mosaic of different habitat types, such as broad-leaved, coniferous, and mixed forests as well as human land-use areas, we assumed that detection probability may change depending on the species’ seasonal habitat preferences. We also accounted for the sampling effort (number of days that a camera trap was functioning) in the detection probability. We showed that overall the detection probabilities were below 0.1 and did not differ among habitat types. The similarity of the detection probabilities across species and habitat types indicates that the main driver of the species’ detection probability is food availability, which is homogeneous across habitat types in the study area (Soyumert et al., 2019). This result also includes human land-use areas, such as farmlands and settlements that lead to alternative food resources for wild mammals, such as livestock, poultry, and crops (e.g., Macdonald and Reynolds, 2004; Smith et al., 2004; Mech and Boitani, 2010;  Morelle and Lejeune, 2015; Ertürk, 2017). We expected seasonal differences in the detection probabilities, because the life cycles differ among species. However, detection probability did not  differ between the seasons. In some species, such as the hare and the brown bear, the detection probability was slightly higher in winter-to-summer transitions than summer-to-winter transitions. The increase in detection probability in winter-to-summer transitions can be a result of increasing food availability in summer. Species adapt their life cycles to fluctuations in food availability. For example, the brown bear’s inactive period ends when food availability increases (Linnell et al., 2000). However, even though we observed these slight differences in the detection probability, none of them were significant due to the overlapping 95% Bayesian credible intervals (BCIs). Especially in the lynx, the 95% BCIs were large compared with the other species (Fig. S10F; Table S2). The lynx is a shy species and tends to roam wide ranges in search for food and shelter, which might have lowered its detection rate (Breitenmoser, 2000; Obbard et al., 2010). Also, the lynx population size is assumed to be small in the area, which may also lower the detection rate (Broadley et al., 2019; Soyumert et al., 2019). With more data from the ongoing survey, the BCIs in this species will likely narrow; that is, the accuracy of the detection rates should improve.


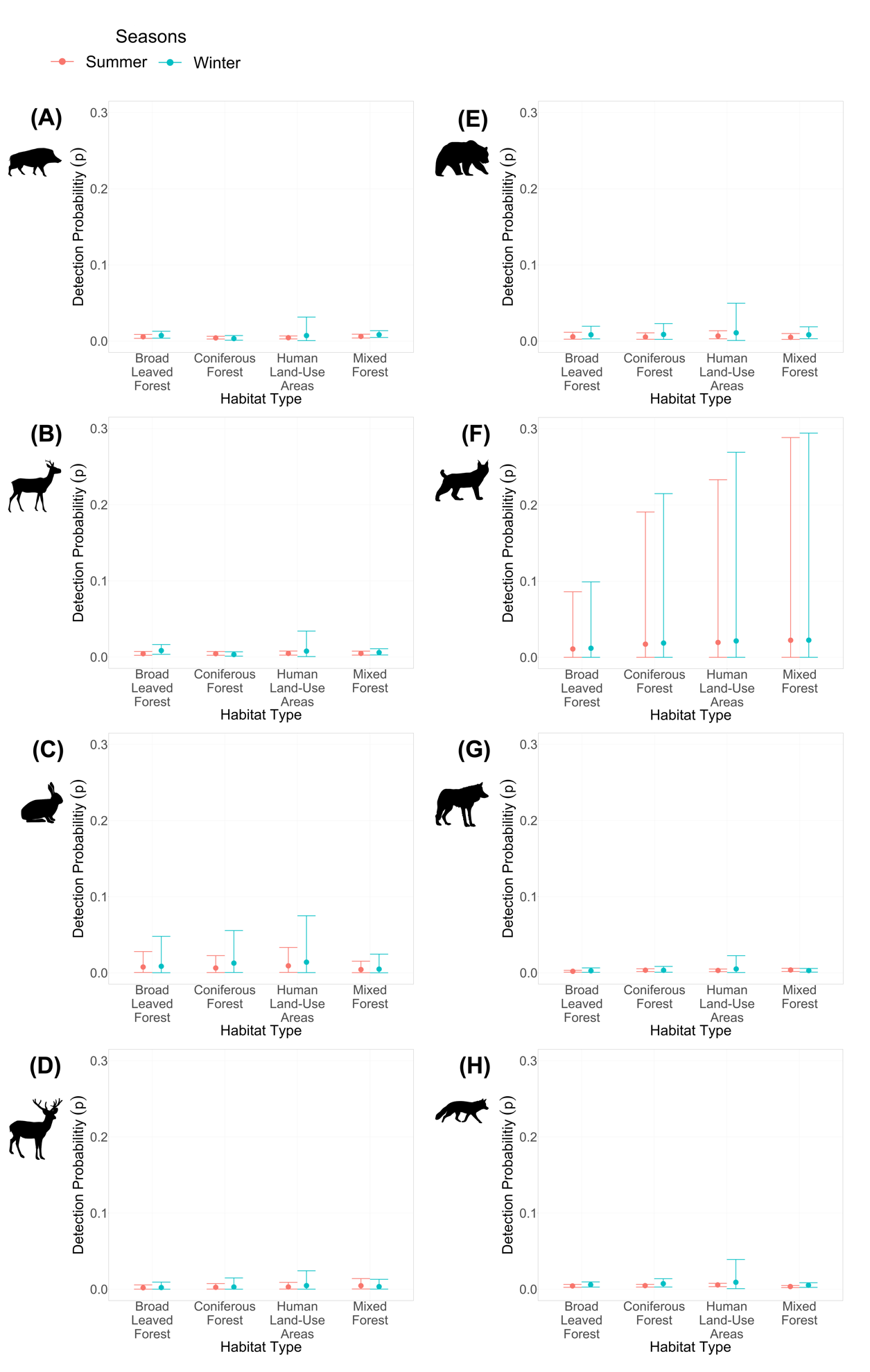


**Fig. S10.** Seasonal detection probabilities of the study species in different habitats. The left column shows the prey species wild boar (A), roe deer (B), European hare (C), and red deer (D); the right column shows the predator species brown bear (E), Eurasian lynx (F), gray wolf (G), and red fox (H). Error bars represent the 95% Bayesian credible intervals.

## References

Breitenmoser, U. (2000). Action plan for the conservation of the Eurasian lynx in Europe (*Lynx lynx*) (No. 18-112). Council of Europe.

Broadley, K., Burton, A. C., Avgar, T., & Boutin, S. (2019). Density-dependent space use affects interpretation of camera trap detection rates. *Ecology and Evolution*, 9(24), 14031-14041. https://doi.org/10.1002/ece3.5840

Ertürk, A. (2017). Research on the spatial ecology and population structure of Anatolian *Canis lupus* L. 1758 (gray wolf). PhD thesis, Hacettepe University, Ankara, Turkey.

Linnell, J. D., Swenson, J. E., Andersen, R., & Barnes, B. (2000). How vulnerable are denning bears to disturbance?. *Wildlife Society Bulletin*, 400-413.

Macdonald, D. W. and Reynolds, J. C. (2004). Red fox *Vulpes vulpes*. — In: Sillero-Zubiri, C. et al. (eds), Canids: foxes, wolves, jackals and dogs. Status Survey and Conservation Action Plan. IUCN/SSC Canid Specialist Group, pp. 129–136.

Mech, L. D., & Boitani, L. (Eds.). (2010). Wolves: behavior, ecology, and conservation. University of Chicago Press.

Morelle, K., & Lejeune, P. (2015). Seasonal variations of wild boar Sus scrofa distribution in agricultural landscapes: a species distribution modelling approach. *European Journal of Wildlife Research*, *61*(1), 45-56. https://doi.org/10.1007/s10344-014-0872-6

Obbard, M. E., Howe, E. J., & Kyle, C. J. (2010). Empirical comparison of density estimators for large carnivores. *Journal of Applied Ecology*, 47(1), 76-84. https://doi.org/10.1111/j.1365-2664.2009.01758.x

Smith, R. K., Jennings, N. V., Robinson, A., & Harris, S. (2004). Conservation of European hares *Lepus europaeus* in Britain: is increasing habitat heterogeneity in farmland the answer?. *Journal of Applied Ecology*, *41*(6), 1092-1102. https://doi.org/10.1111/j.0021-8901.2004.00976.x

Soyumert, A., Ertürk, A., & Tavşanoğlu, Ç. (2019). The importance of lagomorphs for the Eurasian lynx in Western Asia: results from a large scale camera-trapping survey in Turkey. *Mammalian Biology, 95(1),* 18-25. doi:10.1016/j.mambio.2019.01.003
